# Composting dairy cattle manure significantly reduces antibiotic-resistant bacteria with no biochar impact

**DOI:** 10.1128/aem.01258-26

**Published:** 2026-08-13

**Authors:** Getahun E. Agga, Annesly Netthisinghe, Paul Woosley, William Strunk, Karamat R. Sistani

**Affiliations:** 1 Food Animal Environmental Systems Research Unit, Agricultural Research Service, U.S. Department of Agriculture, Bowling Green, Kentucky, USA; 2 Department of Agriculture and Food Science, Western Kentucky University 1889, Bowling Green, Kentucky, USA; University of Nebraska-Lincoln, Lincoln, Nebraska, USA

**Keywords:** manure, biochar, composting, antimicrobial resistance, 3GC^r^, ESBL

## Abstract

In two randomized controlled field studies, we investigated the effect of biochar-amended dairy manure composting on the concentrations and prevalence of generic and antibiotic-resistant *E. coli* and enterococci. Fresh dairy manure mixed with sawdust and biochar was randomly distributed into plastic reactors and composted for 6 months. Pre-compost (biochar, sawdust, fresh dairy manure), manure mixture, and final compost samples were cultured for enumeration and prevalence. While biochar and sawdust were not significant sources, fresh dairy manure was the major contributor of bacteria to the initial manure mixture. Biochar amendment did not significantly reduce bacterial load beyond that could be achieved by composting alone. Spring-summer composting was more effective than fall-winter composting. While *E. coli* was more prone, enterococci were more likely to survive composting. Dairy manure composting eliminated bacteria (3rd generation cephalosporin-resistant [3GC^r^] and extended spectrum beta-lactamase-producing [ESBL]-*E. coli*) resistant to critically important and highest priority antibiotics for human health and significantly reduced the prevalence and concentrations of other investigated bacteria. 3GC^r^- and ESBL-*E. coli* from fresh dairy manure were multidrug resistant and carried *bla*_CTX-M_ genes, a widely distributed ESBL gene type. By whole-genome sequencing, ESBL-*E. coli* from experiment 1 (fall-winter) had diverse resistance profiles, sequence types, plasmid profiles, virulence factor genes, and more *bla*_CTX-M_ genes (CTX-M-1, CTX-M-32, and CTX-M-55). However, those from experiment 2 (spring-summer) were clonal and carried *bla*_CTX-M-55_ only. In conclusion, dairy manure composting effectively removes antibiotic resistant bacteria of public health importance, with no added effect of biochar amendment.

**IMPORTANCE:**

Composting is a microbially mediated decomposition of organic matter into a nutrient-rich product for use as fertilizer. Biochar, obtained from the pyrolysis of biomass including animal manure, has been used for soil amendment. Beyond its nutrient stabilizing potential, composting has been shown to reduce bacterial load in animal manure. However, its effect with or without a biochar addition was not evaluated under a randomized controlled field trial. Biochar was nearly bacteriologically “sterile,” and the sawdust used as a bulking agent had low concentrations of innocuous bacteria indicating that dairy manure was the main source of bacteria to the pre-compost mixture. Biochar did not have an extra effect on the bacterial load beyond composting alone. Composting during spring/summer is more effective than fall/winter. Furthermore, it is more effective against gram-negatives than gram-positive bacteria. Importantly, composting effectively eliminated multidrug-resistant bacteria of critical importance to public health for safer use of dairy manure.

## INTRODUCTION

Land application of untreated animal manure including dairy manure (mixture of feces, urine, and bedding material) is a significant route for the spread of antimicrobial-resistant bacteria including foodborne pathogens (1) by their direct transfer or by enriching resident bacteria in the soil (2–4). Animal manure treatment technologies such as composting may reduce the spread of antimicrobial-resistant bacteria while converting animal manure into a more valuable and safer-to-use products (1, 5). Composting is a self-heating decomposition and stabilization of organic matter through the action of microorganisms under aerobic conditions to produce a nutrient-rich product (compost) from animal manure for use as soil fertilizer for crop production (5–7). However, the effect of composting with respect to reducing antimicrobial-resistant bacteria has not been fully evaluated. First, the majority of published studies focused on evaluating the effects of composting on antimicrobials and antimicrobial resistance genes mostly under laboratory settings, reporting percent reductions often with mixed results (6, 8). Second, mitigating antimicrobial resistance is not the objective of current animal manure composting (1). Therefore, field studies are required to assess the true impact of composting for safe utilization of animal manure while reducing the spread of foodborne pathogens, including antimicrobial-resistant strains, into the environment and eventually to humans.

Biochar is obtained from the pyrolysis of biomass such as animal manure and forest products under low oxygen level (9). As such, biochar is a precursor for activated carbon and has been shown to reduce pathogenic bacteria and their resistance genes in the soil (6). Moreover, biochar can provide nutrition and surface area (6), which may support the growth of bacteria responsible for the degradation of organic matter during composting. Biochar can be used as a bulking agent in a similar way as a source of activated carbon in animal manure composting by also balancing C:N ratio (7).

Beta-lactams (penicillin, 1st and 3rd generation cephalosporins) and tetracyclines are the most common antibiotics used for the prevention and treatment of bacterial infections in U.S. dairy cattle production (1, 10). *E. coli* and enterococci are commonly used as indicators for antibiotic selective pressure in antimicrobial resistance studies (2, 3, 11). Tetracycline resistance (TET^r^) both in Gram-negative and Gram-positive bacteria can be used to evaluate the effectiveness of interventions due to its common occurrence, perhaps due to its historically long-term use in animal production, its dissemination in the environment, and the presence of multiple genetic mechanisms conferring resistance (12, 13). Resistance to beta-lactams is mediated by extended spectrum beta-lactamases (ESBLs) or AmpC beta-lactamases which hydrolyze the β-lactam ring of the antibiotics. ESBLs confer resistance to penicillin and extended-spectrum cephalosporins (third and higher generations), while AmpC β-lactamases confer resistance to third-generation cephalosporins (3GC^r^) (13).

We hypothesized that biochar (BC) dairy manure amendment may enhance composting effectiveness as a preharvest food safety measure to reduce foodborne and antimicrobial-resistant bacteria. In a randomized field trial (7), we evaluated the impact of increased concentrations of biochar for the removal of aminopyralid herbicide residue from dairy manure compost. The objective of the current study was to investigate if biochar amendment of dairy manure enhances the beneficial effect of removing bacteria including antimicrobial-resistant populations, beyond composting alone. For this study, we specifically investigated the effect of dairy manure composting with or without biochar amendment on generic and antibiotic-resistant (TET^r^, 3GC^r^, and ESBL-producing) *E. coli* and generic and TET^r^ enterococci.

## MATERIALS AND METHODS

### Experimental setup

**Fig 1:**
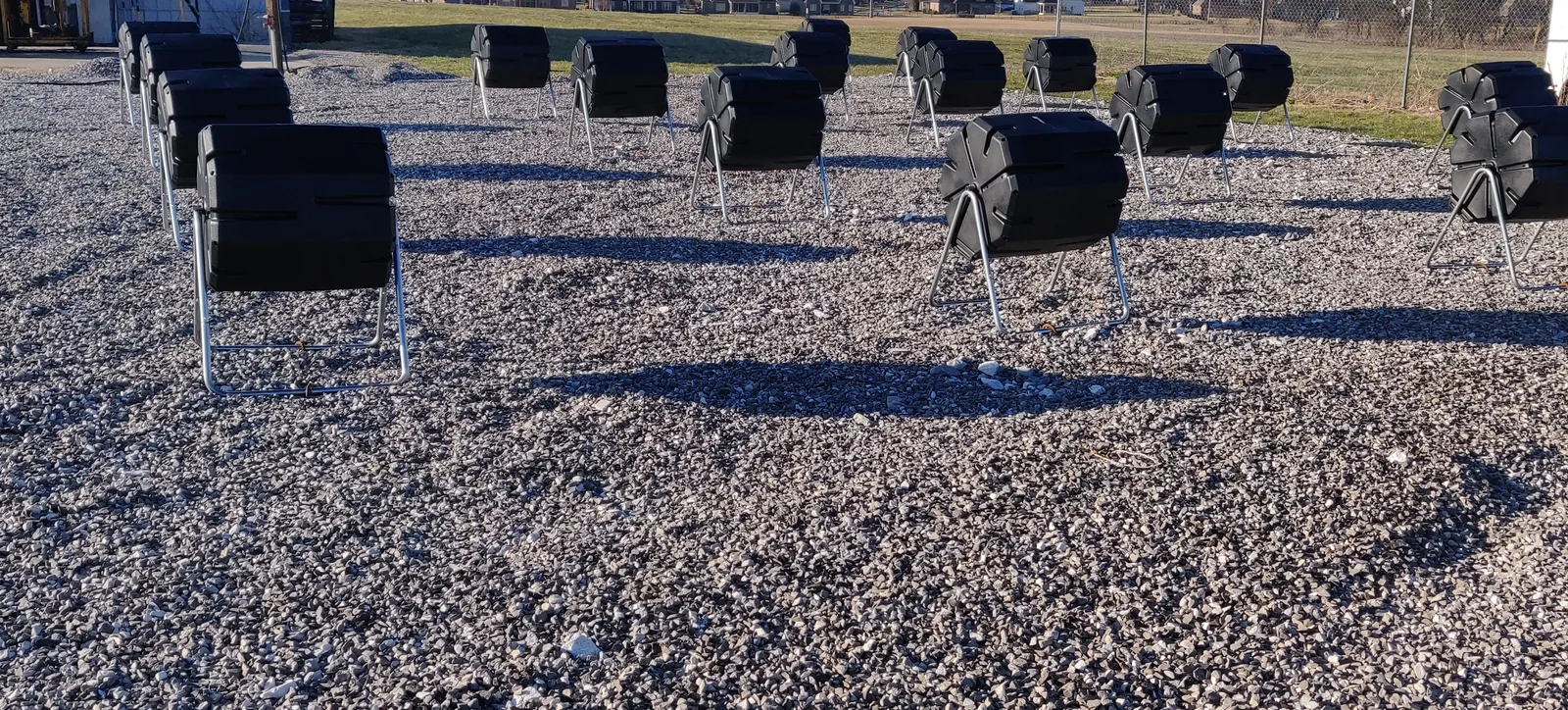
Picture showing a partial setup of field experiments for dairy manure composting with or without biochar.

Two independent randomized composting trials, each lasting for 6 months, were conducted from August 2022 to March 2023 (Experiment 1), roughly representing fall-winter season, and March 2023 to September 2023 (Experiment 2), representing spring-summer season, at Agriculture Research and Education Complex (AREC), Western Kentucky University. Detailed experimental setup and conditions were previously reported (7), and a picture showing a partial setup is shown in Fig. 1. The various treatment levels are summarized in Table 1. Briefly, compost starter mixtures were prepared by mixing 22.5 kg of fresh dairy manure (86%–88% moisture) with an equal amount of sawdust consisting of 8.2%–8.8% moisture, 1.2 g N/kg, 385.2 g C/kg, and 355:1 C/N ratio. Dairy manure was obtained from the manure pit collected from AREC dairy barn that consisted of 53 Holstein Friesian milking cows managed in a free stall barn setting.

**TABLE 1:** Summary of treatment setup for field scale dairy manure composting^*a*^

Experiment	Biochar level (% wt/wt)	Aminopyralid	No. of reactors
Experiment 1	0%	Yes	4
	2%	Yes	4
	4%	Yes	4
	10%	Yes	4
Experiment 2	0%	Yes	4
		No	1
	2%	Yes	4
		No	1
	4%	Yes	4
		No	1
	10%	Yes	4
		No	1

^*a*^ In Experiment 1, all treatments (defined by % biochar) received aminopyralid at 10 ppb. In Experiment 2, one reactor per % biochar did not receive aminopyralid.

The compost starter mixtures (45 kg) were mixed with 0%, 2%, 4%, or 10% (wt/wt) biochar (BC). The BC was obtained from Biochar Now (Berthoud, CO, USA). The compost starter mixtures of the four BC treatments were randomly distributed into 140 litter plastic rotary drum reactors (16 in experiment 1, four reactors per BC treatment; 20 in experiment 2, five reactors per BC treatment) and composted under natural environmental conditions. To aerate and homogenize the material and to promote composting, the reactors were rotated twice in the first week and thereafter once a week. Rotating the reactors was stopped when the temperature inside the compost dropped slowly to reach the ambient temperature. The optimum 50%–60% moisture content (14) was maintained by adding water. Weekly temperature measurements were obtained for the first 4 months using a hand-held 0.9-m probe (Reotemp, San Diago, CA, USA). The two experiments were conducted similarly with one exception: whereas all BC treatment groups (defined by percent biochar) received 10 ppb aminopyralid in Experiment 1, four reactors with no aminopyralid were added in Experiment 2 for each %BC treatment.

### Sample collection

In Experiment 1, the individual constituents including biochar (*n* = 12), sawdust (*n* = 16), and raw manure (*n* = 16) samples (representing 4 samples per %BC treatment) were collected just before they were mixed and added to the reactors. In Experiment 2, only a single bulk raw dairy manure was collected and processed since biochar and sawdust did not contribute significantly to the concentrations of the bacterial strains, and that all the raw dairy manure samples were positive in Experiment 1. Starter mixture samples from Experiment 1 (*n* = 16) and Experiment 2 (*n* = 20) were obtained at the baseline after the mixtures were distributed to the reactors. Final compost samples were collected from Experiment 1 (*n* = 16) and Experiment 2 (*n* = 20) at the end of the 6-month composting cycles. Contents were homogenized, and grab samples were obtained using gloved hands changing gloves between reactors. Samples were collected into individual sealable zip-lock bags and kept in icebox, transported to the lab, and processed on the same day.

### Bacterial culture for enumeration and prevalence

Ten grams of the samples were transferred into a sterile filter barrier bag (Nasco Whirl-Pack) and suspended in 90 mL of buffered peptone water (BPW; Becton, Dickinson and Company [BD], Franklin Lakes, NJ, USA) and homogenized by hand massaging from which 8 mL aliquot was removed for enumeration. Bacteria were enumerated from 50 μL of appropriate BPW serial dilutions of the aliquots using an Eddy Jet 2 spiral plater (Neutec Group Inc., Farmingdale, NY, USA).

Generic (isolated on non-antibiotic supplemented media) *E. coli* was enumerated on modified membrane thermo-tolerant *E. coli* (mTEC) plates (BD) with no antibiotic supplementation. Third-generation cephalosporin-resistant (3GC^r^) *E. coli* and TET^r^ *E. coli* were enumerated on CHROMagar *E. coli* (CEC, DRG International, Springfield, NJ, USA) plates supplemented with 4 µg/mL cefotaxime (CEC + CTX) or 16 µg/mL tetracycline (CEC + TET), respectively. *E. coli* plates were incubated at 37°C for 24 h, as described before (2). Generic enterococci and TET^r^ enterococci were enumerated on Salentz Bartley Medium (SBM) agar (Thermo Fisher Scientific, Waltham, MA, USA), and SBM was supplemented with 16 µg/mL tetracycline (SBM + TET). Enterococcal plates were incubated at 35°C for 4 h and then at 44°C for 48 h, as previously described (3, 15). After incubation, typical colonies on the respective agar types were counted using SphereFlash automated colony counter (Neutec Group Inc.).

To determine bacterial prevalence, the remaining BPW sample suspension was incubated at 25°C for 2 h followed by 42°C for 6 h and then held at 4°C until secondary enrichments were performed the following day as described (2). BPW pre-enrichments were swabbed and streaked onto mTEC, CEC + CTX and CEC + TET for the detection of generic-, 3GC^r^-, and TET^r^-*E. coli* strains, respectively. SBM and SBM + TET plates were used for the detection of generic and TET^r^ *Enterococcus* strains, respectively. For the detection of extended-spectrum beta-lactamase (ESBL)-producing *E. coli*, BPW pre-enrichments were swabbed and streaked onto CHROMagar ESBL plates (DRG International). All prevalence plates were incubated at 37°C for 24 h.esb

Tetracycline and cefotaxime antibiotics were obtained from Millipore Sigma (St. Louis, MO, USA) and added to culture media at their Clinical and Laboratory Standards Institute (CLSI) resistance breakpoint concentrations of ≥16 µg/mL and ≥4 µg/mL, respectively (16). From each enumeration or prevalence plate, two presumptive colonies per positive sample were picked and inoculated into 96-deep well plates (VWR, Radnor, PA, USA) containing tryptic soy broth (TSB; BD) and incubated at 37°C for 24 h. After an aliquot of the broth culture was taken for DNA extraction, plates were stored at −20°C in 15% glycerol (vol/vol) for further analyses. From the CHROMagar ESBL plates, only red colonies were picked for the isolation of ESBL-producing *E. coli*.

### DNA extraction, bacterial confirmation, and species identification

A 10 μL aliquot from fresh TSB culture was combined with BAX lysis buffer (DuPont Qualicon, Inc., Wilmington, DE, USA) for DNA extraction according to the manufacturer’s instructions. Presumptive *E. coli* isolates of the four strain types (generic, TET^r^, 3GC^r^, and ESBL-producing *E. coli*) were confirmed by PCR using published primers and protocols (17). Confirmation and species identification of *Enterococcus* isolates were conducted by matrix-assisted laser desorption ionization-time-of-flight mass spectrometry (MALDI-TOF; Biotyper, Bruker Daltonics, Billerica, MA, USA) at the National Veterinary Services Laboratories, Iowa, USA.

### Antimicrobial susceptibility testing

One isolate per positive sample from antibiotic-resistant *E. coli* (3GC^r^ *E. coli*, ESBL-producing *E. coli*, and TET^r^ *E. coli*), and generic and TET^r^ *Enterococcus* isolates were tested for their antimicrobial resistance by broth microdilution using the Sensititre system (Thermo Scientific Microbiology, Oakwood Village, OH, USA). *E. coli* isolates were tested against 14 antimicrobials on the National Antimicrobial Resistance Monitoring System (NARMS) Gram-negative (CMV4AGNF) plates (Thermo Fisher Scientific). The antimicrobials represented 10 antimicrobial classes (Table S1) including aminoglycosides (gentamicin and streptomycin), penicillins (ampicillin), cephems (cefoxitin and ceftriaxone), β-lactam combination agents (amoxicillin + clavulanic acid), penems (meropenem), folate pathway antagonists (sulfisoxazole and trimethoprim + sulfamethoxazole), macrolides (azithromycin), phenicols (chloramphenicol), quinolones (ciprofloxacin and nalidixic acid), and tetracyclines (tetracycline). 3GC^r^ *E. coli* isolates (*n* = 18) obtained from the second experiment were also tested using the CMV5AGNF plates that included polymyxin (colistin) instead of streptomycin.

*Enterococcus* isolates were tested against 14 antimicrobials on the NARMS Gram-positive (CMV4AGP) plates (Thermo Fisher Scientific) representing 13 classes (Table S2) including aminoglycosides (gentamicin and streptomycin), glycopeptides (vancomycin), glycylcycline (tigecycline), lipopeptides (daptomycin), macrolides (erythromycin), nitrofurans (nitrofurantoin), orthosomycin (avilamycin), oxazolidinones (linezolid), penicillins (ampicillin), phenicols (chloramphenicol), quinolones (ciprofloxacin), streptogramins (quinupristin-dalfopristin), and tetracyclines (tetracycline). Minimum inhibitory concentration (MIC) of the antimicrobials was determined for each isolate according to CLSI guidelines. *E. coli* ATCC 25922 and *E. faecalis* ATCC 29212 were used as quality-control strains.

Results were interpreted using the CLSI breakpoints to classify the isolates as susceptible, intermediate, or resistant, where available (16). For antibiotics with no CLSI breakpoints, NARMS consensus breakpoints were used. Isolates resistant to ≥3 antimicrobial classes based on CLSI classification system (16) were considered multidrug-resistant (MDR) (18). Intermediate values were reclassified as susceptible to create a binary outcome of susceptible and resistant to compare resistance prevalence by the level of biochar amendment.

### Detection of antimicrobial resistance genes

Phenotypically resistant *E. coli* isolates (3GC^r^, ESBL-producing*,* and TET^r^ *E. coli*) were tested by PCR for their carriage of ARGs as described (13). 3GC^r^ *E. coli* and ESBL-producing *E. coli* isolates were tested for the beta-lactamase genes *bla*_CMY-2_ and *bla*_CTX-M_ genes in duplex PCR. To identify specific subtypes, *bla*_CTX-M_ positive isolates were tested by multiplex PCR assays using ARM-D β-lactamase kit (Streck, La Vista, NE, USA). TET^r^ *E. coli* isolates were tested for tetracycline resistance genes *tet*(A) and *tet*(B) in duplex PCR. TET^r^ enterococci were tested first for *tet*(M), and negative isolates were tested for other *tet* genes (L, O, and P) as previously described (15). Primers were obtained from Integrated DNA Technologies, Inc. (IDT; Coralville, IA, USA). Amplification products were analyzed by capillary gel electrophoresis (QIAxcel Fast Analysis System, Qiagen) using 100 bp DNA ladder (Thermo Fisher Scientific).

### Whole-genome sequencing

Since we observed stark differences between the two composting events with respect to their quinolone susceptibility test profiles of the *E. coli* isolates, 10 isolates (each of the 3GC^r^ *E. coli* and ESBL-producing *E. coli*) with five isolates each from both experiments were whole genome sequenced for further analysis. The 3GC^r^ *E. coli* and the ESBL-producing *E. coli* isolates selected for whole-genome sequencing (WGS) from experiment 1 had either intermediate and/or susceptible classification for ciprofloxacin (MIC ≤ 0.5) and nalidixic acid (MIC ≤ 16), the two quinolone drugs on the NARMS plate. On the other hand, isolates selected from experiment 2 were all resistant to ciprofloxacin (MIC > 4) and nalidixic acid (MIC > 32). Sequencing was done by the USDA-National Veterinary Services Laboratories, Ames, IA. DNA was extracted from an isolated bacterial colony using Promega Maxwell Whole Blood DNA kit (Thermo Fisher Scientific). Library prep was performed using Illumina’s Nextera XT DNA library preparation (Illumina, San Diego, CA, USA), and DNA was sequenced using MiSeq 2 × 250 read generation on an Illumina platform. Sequence depth was calculated by number of reads × 240/Genome length. Genome length was obtained based on multilocus sequence type (MLST) identification. *De novo* assembly was performed using SPAdes genome assembler v3.15.5. MLST data were obtained using 2.23.0 software at the following website: https://github.com/tseemann/mlst. Information on MLST schemes and allelic profiles can be found at https://pubmlst.org/. Antimicrobial resistance genes (ARGs) were identified using AMRFinder, which uses BLASTX to search for a hierarchy of gene families with predetermined cutoffs. Plasmids were identified by using PlasmidFinder-2.0 for Enterobacteriales from the raw sequencing reads in fastq formats (19). Virulence factors were identified using VirulenceFinder 2.0 for *E. coli* from raw sequencing reads in fastq format (20).

### Statistical analysis

Enumeration data, expressed as the number of CFUs/mL, were transformed into log_10_ and analyzed by analysis of variance (ANOVA). In experiment 1, data from the individual components (biochar, sawdust, and raw dairy manure) were compared by treatment group (i.e., the level of biochar amendment) to ensure lack of differences at the baseline which was expected by the randomization process. For experiment 2, we evaluated the effect of aminopyralid on bacterial concentrations. Subsequently, data were pooled by sample type for statistical comparisons if the differences were not statistically significant. For both experiments, we evaluated the effect of biochar amendments and composting on the bacterial concentrations in the manure mixture and final compost. Differences in bacterial concentrations in the raw manure, manure mixture, and final compost between the two experiments were evaluated and combined if they did not significantly differ. Fisher’s exact Chi square test was used to evaluate the effects of experiment (1 vs 2) and %BC on the prevalence (defined as a binary outcome expressed as presence or absence) of the tested bacteria since the outcomes were either sparse or saturated (close to 100%). Manure mix, Experiment 1, 0% biochar, and no aminopyralid groups served as the reference categories for comparing bacterial concentrations among individual components, assessing the effect of composting (experimental differences), and evaluating the impacts of biochar amendments and aminopyralid treatment, respectively. Data were analyzed in STATA 19 (Stata Corp., College Station, TX, USA).

## RESULTS

### Biochar and sawdust added during pre-composting did not increase the bacterial load of dairy manure

**Fig 2:**
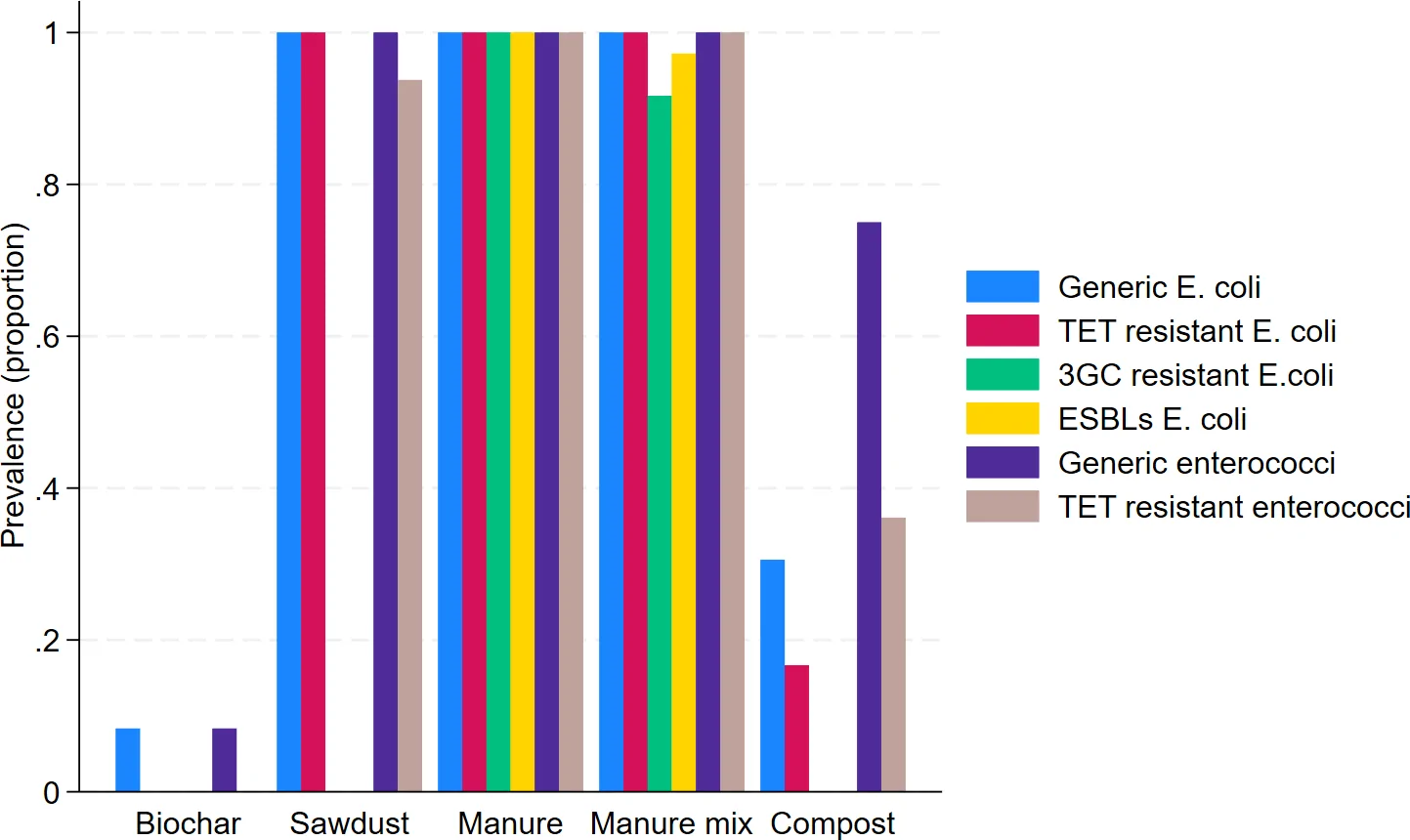
Prevalence of generic and antibiotic-resistant *E. coli* and enterococci in pre-compost samples. Prevalence results are given as bar charts aggregated by sample type: biochar (*n* = 12), sawdust (*n* = 16), manure (*n* = 17), manure mix (*n* = 36), and compost (*n* = 36).

**Fig 3:**
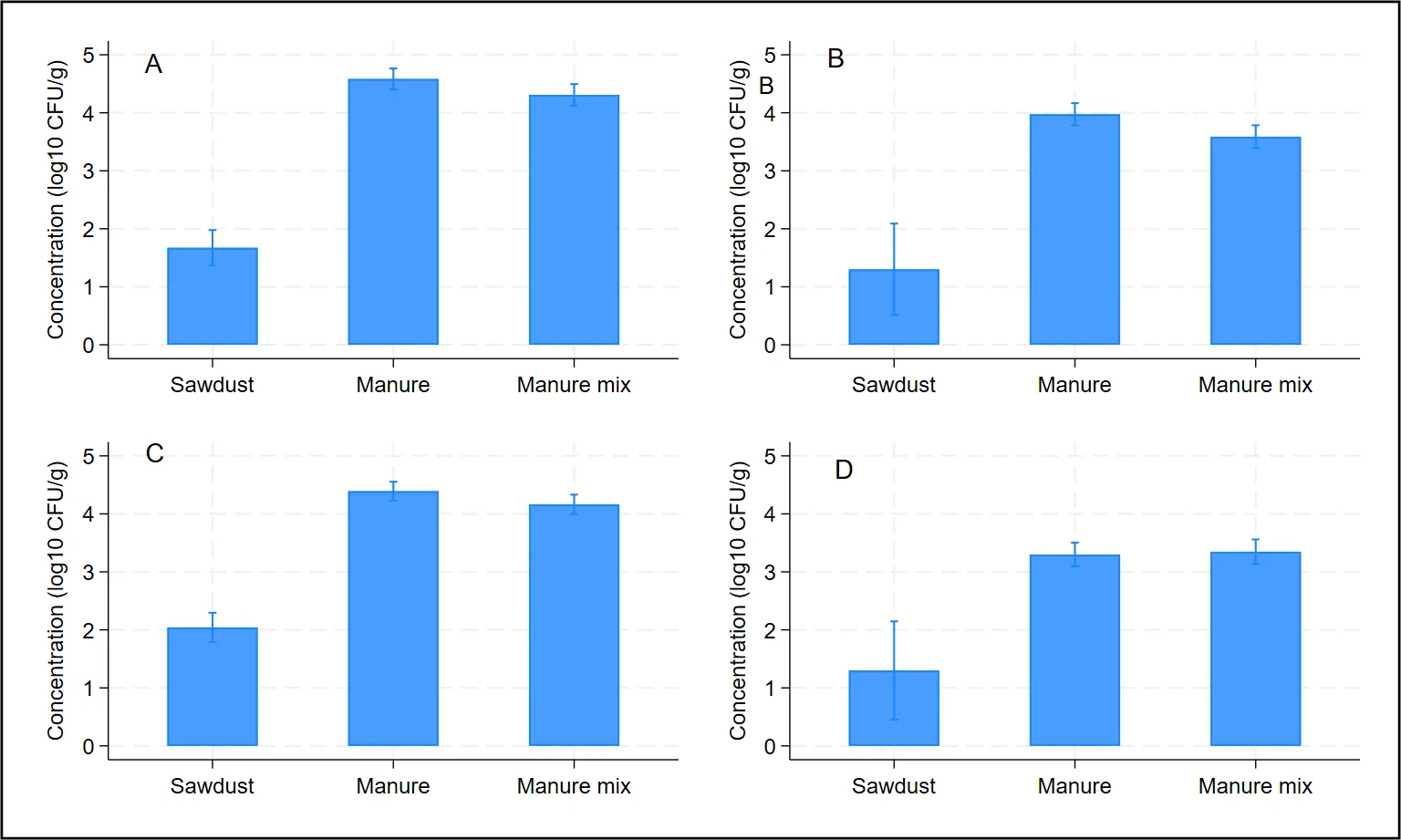
Model predicted concentrations of generic and tetracycline-resistant *E. coli* and enterococci in pre-compost samples. (**A**) Generic *E. coli*; (**B**) generic enterococci; (**C**) tetracycline-resistant *E. coli*; (**D**) tetracycline-resistant enterococci. Values are given as mean concentrations (log_10_ CFU/g) and their 95% confidence intervals.

Overall prevalence (i.e., after secondary enrichment) of generic and antibiotic-resistant *E. coli* and enterococci from pre-compost and compost samples is shown in Fig. 2. Biochar showed almost no detectable bacteria (only a single biochar sample was positive for both generic *E. coli* and enterococci; *n* = 12), while all sawdust samples were positive for generic *E. coli*, TET^r^*E. coli*, and generic enterococci, and 94% (*n* = 16) were positive for TET^r^ enterococci. All raw manure samples (*n* = 17) were positive for all tested bacterial groups, including resistant strains. Because bacterial mean concentrations did not significantly differ (*P* > 0.05) by biochar percentage, data were pooled, revealing that sample type significantly influenced (*P* < 0.0001; Fig. 3) bacterial levels: sawdust had the lowest mean concentrations, whereas manure and manure mix had consistently high levels of generic and resistant bacteria. Although generic *E. coli,* generic *Enterococcus*, and TET^r^ *Enterococcus* mean concentrations were similar (*P* > 0.05) between manure and manure mix, manure had significantly higher mean concentration of TET^r^ *E. coli*, and only manure contained enumerable concentrations of 3GC^r^ *E. coli*. ESBL-producing *E. coli* were not enumerable in any sample type. Additional detailed results are provided in the supplemental material.

### Effect of biochar amended dairy manure composting on concentrations and prevalence of generic and antibiotic-resistant bacteria

**Fig 4:**
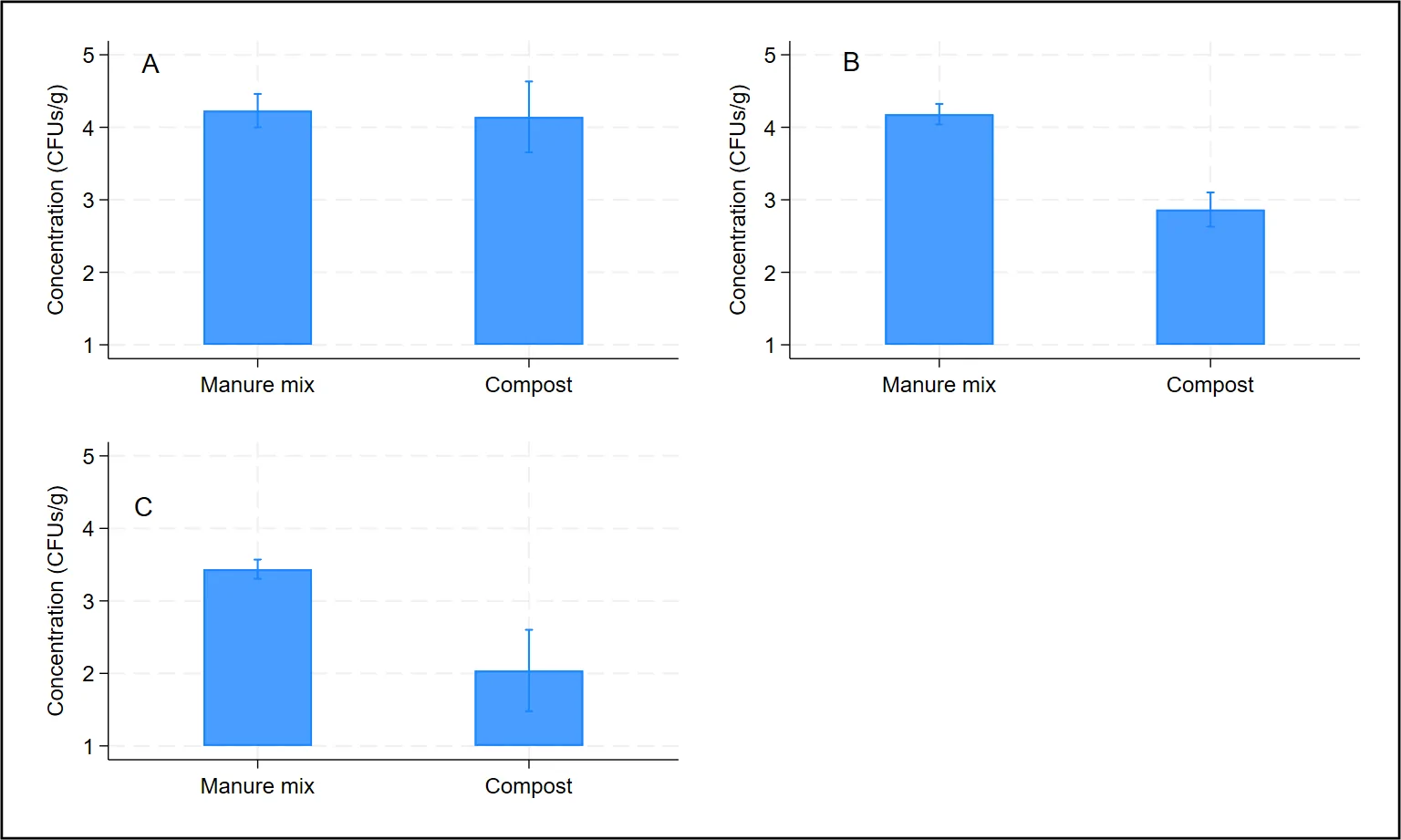
Effect of composting and biochar treatment on the concentration (log_10_ CFU/g) of *E. coli* and enterococci. (**A**) Generic *E. coli* concentration; (**B**) generic *Enterococcus* concentration; (**C**) tetracycline-resistant *Enterococcus* concentration.

Aminopyralid treatment did not significantly affect (*P* > 0.05) mean concentrations or prevalence of generic or antimicrobial-resistant *E. coli* and enterococci in manure mixtures or compost. Because aminopyralid had no measurable effect, data were combined across treatments to evaluate composting and biochar effects. Mean concentrations of generic and TET^r^ *E. coli* and enterococci in manure mixture and final compost by the level of biochar amendment are shown in Table 2. Biochar did not significantly affect (*P* > 0.05) mean concentrations or prevalence of generic *E. coli*, TET^r^ *E. coli*, generic enterococci, or TET^r^ enterococci in the composted material. However, the highest biochar level (10%) appeared to enhance reductions, as nearly all compost samples at this concentration were free of enumerable bacteria in experiment 1, except for three samples which were enumerable for generic enterococci. Composting significantly (*P* < 0.0001) reduced the mean concentrations of generic enterococci and TET^r^ enterococci when compared to manure mixture (Fig. 4). Overall, composting—rather than aminopyralid or biochar—was the primary driver of reductions in both concentrations and prevalence of generic and antimicrobial-resistant bacteria in dairy manure. More importantly, composting effectively removed 3GC^r^ *E. coli* and ESBL *E. coli,* which are resistant to critically important antibiotics for human use. Further detailed results are provided in the supplemental material.

**TABLE 2:** Mean concentrations of bacteria before and after composting of dairy manure amended with various concentrations of biochar

Bacterial type	Percent biochar	Manure mixture				Compost			
		Mean^*a*^	Standard error	95% confidence interval		Mean^*a*^	Standard error	95% confidence interval	
Generic *E. coli*	0%	4.1	0.1	3.8	4.4	4.7 (2/9)	1.1	2.1	7.3
	2%	4.3	0.1	4.0	4.5	4.5 (2/9)	0.8	2.7	6.3
	4%	4.2	0.3	3.6	4.8	3.7 (4/9)	0.5	2.4	4.9
	10%	4.4	0.2	4.0	4.7				
	Overall	4.2	0.1	4.0	4.5	4.1	0.4	3.7	4.6
Generic enterococci	0%	4.2	0.0	4.1	4.3	3.0 (4/9)	0.4	2.1	3.8
	2%	4.1	0.1	4.0	4.3	3.5 (2/9)	0.8	1.9	5.2
	4%	4.1	0.2	3.8	4.5	2.5 (4/9)	0.1	2.3	2.6
	10%	4.2	0.1	4.1	4.4	2.8 (3/9)	0.2	2.3	3.3
	Overall	4.2	0.1	4.0	4.3	2.9	0.1	2.6	3.1
Tetracycline-resistant *E. coli*	0%	3.5	0.1	3.2	3.7				
	2%	3.6	0.2	3.3	3.9				
	4%^*b*^	3.8 (8/9)	0.1	3.6	4.0				
	10%	3.7	0.2	3.3	4.1				
	Overall	3.6	0.1	3.5	3.8				
Tetracycline-resistant enterococci	0%	3.4	0.1	3.3	3.6	2.0 (2/9)	0.3	−1.3	5.4
	2%	3.4	0.1	3.2	3.6				
	4%	3.4	0.2	3.1	3.8				
	10%	3.5	0.2	3.2	3.8				
	Overall	3.4	0.1	3.3	3.6	2.0	0.3	−1.3	5.4

^*a*^ Numbers in parentheses are the fractions of compost samples that yielded enumerable culture.

^*b*^ One manure mix sample from the 4% biochar was enumeration negative.

### Antimicrobial resistance of 3GC^r^ *E. coli*, ESBL-producing *E. coli,* and TET^r^ *E. coli*

The antimicrobial resistance of the 3GC^r^ *E. coli*, ESBL-producing *E. coli*, and TET^r^ *E. coli* isolates is shown in Table 3. The antimicrobial resistance of the 3GC^r^ *E. coli* isolates to various antimicrobials had a stark difference between the two experiments. The 3GC^r^ *E. coli* isolates from experiment 1 (*n* = 32) had variations with respect to resistance to the drugs tested. Their resistance prevalence varied from 0% (three drugs) to 100% (five drugs). On the other hand, all 3GC^r^ *E. coli* isolates from experiment 2 were either 100% resistant to nine drugs or 100% susceptible to the remaining six drugs. Differences were also observed with respect to resistance to individual drugs. While azithromycin and gentamycin resistance were observed only in the 3GC^r^ *E. coli* isolates from experiment 1 (15.6% and 22%, respectively), they were not detected in any isolates obtained from experiment 2. On the other hand, while all isolates from experiment 2 (*n* = 18) were resistant to ciprofloxacin, nalidixic acid, and trimethoprim/sulfamethoxazole, only 3.1%, 0%, and 12.5%, respectively, of the isolates from experiment 1 (*n* = 32) were resistant to these drugs. The ESBL-producing *E. coli* isolates (*n* = 32) obtained from experiment 1 also had a diverse range of percent resistance to individual antimicrobials than isolates from experiment 2 (Table 3). Isolates from experiment 2 (*n* = 20) were 100% resistant to six drugs and 95% resistant to three drugs. Azithromycin and gentamycin resistance were observed among the isolates from experiment 1, but not in any of the isolates obtained from experiment 2. On the other hand, while 95% of isolates from experiment 2 were resistant to ciprofloxacin, nalidixic acid, and trimethoprim/sulfamethoxazole, isolates from experiment 1 were either all susceptible or had 9.4% of isolates being resistant.

**TABLE 3:** Antimicrobial resistance (%) of third-generation cephalosporin-resistant (3GC^r^) *E. coli*, extended spectrum beta-lactamase (ESBL)-producing *E. coli*, and tetracycline resistant (TET^r^) *E. coli* isolates obtained from fresh and initial mix for dairy manure composting^*a*^

Antimicrobial	3GC^r^ *E. coli*		ESBL-producing *E. coli*		TET^r^ *E. coli*	
	Experiment 1 (*n* = 32)	Experiment 2 (*n* = 18)	Experiment 1 (*n* = 32)	Experiment 2 (*n* = 20)	Experiment 1 (*n* = 38)	Experiment 2 (*n* = 21)
Amoxicillin-clavulanic acid	0	0	3.1	5.0	0	0
Ampicillin	100	100	100	100	36.8	42.9
Azithromycin	15.6	0	12.5	0	0	0
Cefoxitin	3.1	0	0	0	0	0
Ceftriaxone	100	100	90.6	100	0	0
Chloramphenicol	100	100	87.5	100	34.2	23.8
Ciprofloxacin	3.1	100	0	95	0	0
Gentamicin	21.9	0	21.9	0	0	0
Meropenem	0	0	0	0	0	0
Nalidixic acid	0	100	0	95	0	0
Streptomycin	93.8	100	65.6	100	47.4	0
Sulfisoxazole	100	100	87.5	100	36.8	19.1
Tetracycline	100	100	87.5	100	100	100
Trimethoprim-sulfamethoxazole	12.5	100	9.4	95	7.9	0
Colistin (*n* = 18)	NA^*b*^	0	NA	NA	NA	NA

^*a*^ 3GC^r^ *E. coli* isolates (*n* = 18) were also tested with the CMV5AGNF panel, which contains colistin instead of streptomycin.

^*b*^ NA, not applicable.

TET^r^ *E. coli* had low prevalence of co-resistance to few other antimicrobials compared to the 3GC^r^ *E. coli* and ESBL-producing *E. coli* isolates (Table 3). Co-resistance of TET^r^ *E. coli* isolates was observed to combinations of drugs in four antimicrobial classes: penicillins (ampicillin), phenicols (chloramphenicol), aminoglycosides (streptomycin), and folate pathway inhibitors (sulfisoxazole and trimethoprim/sulfamethoxazole). Isolates obtained from the two experiments had similar levels of resistance to three antibiotics; however, there were also important noticeable differences. While streptomycin and trimethoprim/sulfamethoxazole resistance occurred in isolates from experiment 1, they were not detected in isolates from experiment 2.

Resistance profiles of 3GC^r^ *E. coli*, ESBL-producing *E. coli*, and TET^r^ *E. coli* isolates are shown in Table 4. All 3GC^r^ *E. coli* isolates from experiment 1 were MDR by being resistant to 6 (84.4%), 7 (12.5%), or 8 (3.1%) antimicrobial classes. Seven resistance profiles (three in dairy manure and six in dairy manure mix) were identified with 75% of the isolates having a profile containing six classes. On the other hand, all 3GC^r^ *E. coli* isolates from experiment 2 had a similar resistance profile with 100% resistance to nine drugs and 100% susceptibility to the remaining six drugs. Thus, all 3GC^r^ *E. coli* isolated from experiment 2 were MDR by being resistant to seven antimicrobial classes. Also, while all ESBL-producing *E. coli* isolates (*n* = 20) obtained from experiment 2 were MDR being resistant to 6 (5%), 7 (90%), or 8 (5%) antimicrobial classes, only 87.5% of the isolates from experiment 1 were MDR by being resistant to 6 (78.1%) or 7 (9.4%) antimicrobial classes with the remaining 12.5% being either singly resistant to ampicillin (9.4%) or doubly resistant to ampicillin and ceftriaxone (3.1%). Isolates from experiment 1 were more diverse with nine resistance profiles compared to only three profiles observed in the isolates (90% of the isolates had a single profile) obtained from experiment 2. 3GC^r^ and ESBL *E. coli* isolates from the dairy manure mix from experiment 1 had the most diverse resistance profiles than isolates from dairy manure samples. Overall, 44.7% of the TET^r^ *E. coli* isolates (*n* = 38) from experiment 1 were MDR by being resistant to 3 (21.1%), 4 (7.9%), or 5 (15.8%) antimicrobial classes. The remaining isolates (55.3%) were resistant only to tetracycline itself (29%) or one additional drug in other antimicrobial classes (26.3%). In comparison, only 23.8% of the isolates obtained from experiment 2 were MDR by being resistant to 3 (4.8) or 4 (19.1%) classes, with the remaining isolates being resistant only to tetracycline itself (57.1%) or one additional drug in other antimicrobial classes (19.1%). Among the 10 resistance profiles detected, six (five resistance profiles included streptomycin, one included trimethoprim/sulfamethoxazole) were unique to isolates obtained from experiment 1, while the remaining four profiles were shared. The six isolates obtained from the final compost samples from experiment 1 had unique MDR profiles: three isolates had chloramphenicol-sulfisoxazole-tetracycline-trimethoprim/sulfamethoxazole and the other three isolates had streptomycin-sulfisoxazole-tetracycline.

**TABLE 4:** Antimicrobial resistance profiles of third-generation cephalosporin-resistant (3GC^r^) *E. coli,* extended spectrum beta-lactamase-producing (ESBL) *E. coli*, and TET^r^ *E. coli* isolates obtained from dairy manure, manure mix, or final compost^*a*^

Bacterial type	Resistance profile	Experiment 1			Experiment 2		
		Dairy manure	Manure mix	Final compost	Dairy manure	Manure mix	
3GC^r^ *E. coli*	amp_axo_chl_str_sul_tet_	14	10				
	amp_azi_axo_chl_gen_str_sul_tet_tri_	1	1				
	amp_azi_axo_chl_str_sul_tet_	1					
	amp_azi_axo_chl_cip_gen_str_sul_tet_tri_		1				
	amp_azi_fox_axo_chl_gen_str_sul_tet_tri_		1				
	amp_axo_chl_gen_sul_tet_		2				
	amp_axo_chl_gen_str_sul_tet_		1				
	amp_axo_chl_cip_nal_str_sul_tet_tri_				1	17	
	**Total isolates**	**16**	**16**		**1**	**17**	
ESBL *E. coli*	amp_	2	1				
	amp_axo_chl_gen_str_sul_tet_	1					
	amp_axo_chl_gen_sul_tet_	2	3				
	amp_axo_chl_str_sul_tet_	10	7			1	
	amp_azi_axo_chl_sul_tet_tri_	1	1				
	amo_amp_axo_chl_str_sul_tet_		1				
	amp_axo_		1				
	amp_azi_axo_chl_gen_str_sul_tet_tri_		1				
	amp_azi_axo_chl_str_sul_tet_		1				
	amo_amp_axo_chl_cip_nal_str_sul_tet_tri_					1	
	amp_axo_chl_cip_nal_str_sul_tet_tri				1	17	
	**Total isolates**	**16**	**16**		**1**	**19**	
TET^r^ *E. coli*	amp_chl_str_sul_tet_	3	3				
	amp_chl_str_tet_	1					
	amp_chl_sul_tet_	1	1		1	3	
	amp_chl_tet_		1			1	
	amp_str_tet_	1					
	amp_tet_	1	2			4	
	chl_sul_tet_tri_			3			
	str_sul_tet_			3			
	str_tet_	3	4				
	tet_	6	5			12	
	**Total isolates**	**16**	**16**	**6**	**1**	**20**	

^*a*^ 3GC^r^ *E. coli *and ESBL *E. coli *were not detected from any final compost. TET^r^ *E. coli* was detected from six final compost samples in experiment 1. Antimicrobial drug abbreviations: amo, amoxicillin + clavulanic acid; amp, ampicillin; axo, ceftriaxone; fox, cefoxitin; chl, chloramphenicol; gen, gentamicin; str, streptomycin; sul, sulfisoxazole; tet, tetracycline; tri, trimethoprim + sulfamethoxazole; cip, ciprofloxacin; nal, nalidixic acid; azi, azithromycin.

### PCR detection of antimicrobial resistance genes

All 3GC^r^ *E. coli* isolates (*n* = 50; 17 isolates from dairy manure and 33 isolates from manure mix) and ESBL-producing *E. coli* (*n* = 52; 17 isolates from dairy manure and 35 from initial manure mix) were PCR positive for the CTX-M-15 *bla* gene family. TET^r^ *E. coli* were positive either for *tet*(A) or *tet*(B), the distribution of which was significantly (Fisher’s exact *P* = 0.004) affected by sample type. While the majority of dairy manure (76.5%; *n* = 17 isolates) and manure mix (58.3%; *n* = 36) were *tet*(A) positive, all six isolates obtained from final compost samples were *tet*(B) positive.

### Whole-genome sequencing characterization of 3GC^r^ and ESBL *E. coli* isolates

#### WGS quality assessments and statistics

Whole-genome sequence and assembly statistics of 20 *E. coli* isolates are presented in Table S3. Forward (R1) reads had an average read score of 39.1 (*σ* = 0.04; range: 39.0–39.2) and a mean Q30 pass percentage of 98.7% (*σ* = 0.4; range: 98.4%–99%). Reverse reads (R2) had read scores averaging 38.5 (*σ* = 0.12; range: 38.2–38.7) and a mean Q30 pass percentage of 97.0% (*σ* = 0.4; range: 96.5%–98.0%). Mean sequencing depth was 193.8 (*σ* = 23.5; range: 161.4–234.8), and the genome length of the *E. coli* isolates was 5 million bp. The mean number of scaffolds detected was 199.9 (*σ* = 35.6; range: 154–294) with a mean N50 of 201,154 (*σ* = 48,149.33; range: 133,428–275,396). Full description of antimicrobial resistance genes is presented in Table S4.

#### Sequence type variation was dependent on the composting experiment

The 10 isolates obtained from the pre-compost dairy manure mixture samples in the first composting experiment were more diverse with four STs: 10, 189, 515, and 6416. On the other hand, all 10 isolates from the pre-compost dairy manure mixture for the second composting experiment belonged to a single ST 2509.

#### Sequence type-dependent AST, ARG, plasmid, and VFG profiles of ESBL-producing *E. coli* from pre-composting dairy manure mixtures

All isolates obtained on cefotaxime-supplemented media for the prevalence and enumeration of 3GC^r^ *E. coli* from the pre-composting dairy manure mixtures were positive for *bla*_CTX-M_ by PCR. This was confirmed by WGS that all isolates carried *bla*_CTX-M-1_, *bla*_CTX-M-32_, or *bla*_CTX-M-55_. These gene variants belong to the CTX-M-15 gene family on the Streck ARM-D PCR assay kit. Therefore, all 3GC^r^ *E. coli* isolates are also categorized as ESBL-producing *E. coli*.

#### Within ST and between composting experiment variations of AST profiles

AST-, ARG-, plasmid-, and VFG-profiles of 20 ESBL-producing isolates further analyzed by WGS are presented in Table 5. Six different antimicrobial susceptibility test profiles were identified. While isolates from experiment 2 had similar AST profiles, the isolates from experiment 1 had five profiles whose diversity varied within and between the STs (Table 5). All isolates were MDR being resistant to five (eight isolates from composting 1), seven (one ST189 isolate from experiment 1, and all isolates from experiment 2), or eight (one ST189 isolate from experiment 1) antimicrobial classes.

**TABLE 5:** Antimicrobial susceptibility test (AST), antimicrobial resistance gene (ARG), plasmid, and virulence factor gene profiles of 20 extended spectrum beta-lactamase-producing *E. coli* isolates obtained from dairy manure pre-compost mixture samples

ST	Sample ID	AST profile	ARG profile	Plasmid profile	Virulence factor genes
515	1	str amp axo sul chl tet	*aph*(3”)-Ib, *aph*(6)-Id *bla*_CTX-M-1_, *bla*_TEM-1_ *sul*2, *mph*(A), *flo*R, *qnr*S1 *tet*(A), *tet*(M), *glp*T, *uhp*T	IncFIA(HI1) IncHI1A IncHI1B(R27) IncR	*asl*A, *cap*U, *csg*A, *gad*, *hly*E, *nlp*I, *ter*C, *yeh*A, *yeh*B, *yeh*C, *yeh*D
	3				
6416	2		*aph*(3”)-Ib, *aph*(6)-Id *bla*_CTX-M-1_, *bla*_TEM-1_ *sul*2, *mph*(A), *flo*R, *qnr*B19, *tet*(A) *glp*T, *uhp*T	IncR Col(pHAD28)	*asl*A, *fde*C, *hly*E, *nlp*I, *ter*C, *yeh*B, *yeh*C, *yeh*D
10	4	gen str amp axo sul chl tet	*aph*(3’)-Ia, *aac*(3)-IIe, *aad*A1 *bla*_CTX-M-55_ *sul*3, *floR qnr*B19 *tet*(A)	IncFIB(AP001918) Col(pHAD28)	*asl*A, *anr*, *cma*, *csg*A, *fim*H, *gad*, *hly*E, *hly*F, *iss*, *iuc*C, *iut*A, *nlp*I, *sit*A, *ter*C*, traJ*, *tra*T, *yeh*A, *yeh*B, *yeh*C, *yeh*D
	6	gen amp axo sul chl tet			Plus *omp*T
	10				
	8	str amp axo sul chl tet	*aph*(3”)-Ib, *aph*(6)-Id *bla*_CTX-M-32_, *bla*_TEM-1_ *sul*2, *flo*R, *tet*(A)	IncR	*asl*A, *csg*A, *fim*H, *gad*, *hly*E, *nlp*I, *ter*C, *yeh*A, *yeh*B, *yeh*C, *yeh*D, *ygh*J
	9				
189	5	gen str amp axo sul tri azi chl cip tet	*aph*(3’)-Ia, *aac*(3)-IId, *aad*A22 *bla*_CTX-M-55_, *bla*_TEM-1,_*bla*_LAP-2_ *sul*3, *dfr*A14, *mph*(A), *lnu*(F) *flo*R, *qnr*S1, *tet*(A), *tet*(B) *glp*T, *arr*-2	IncHI2 IncHI2A IncN	*asl*A, *csg*A, *fim*H, *gad*, *hly*E, *nlp*I, *ter*C, *ygh*J
	7	gen str amp axo sul tri azi chl tet			
2509	11-20	str amp axo sul tri chl cip nal tet	*aph*(3”)-Ib, *aph*(6)-Id, *aad*A5 *bla*_CTX-M-55_*sul*2, *dfr*A17, *flo*R *gyr*A-D87N, *gyr*A-S83L, *par*C, *parE tet*(A), *glp*T, *nfs*A, *pmr*B	p0111	F17A, F17D, F17G, *csg*A, f*de*C, *fim*H, *gad*, *hly*E*, lpf*A, *nlp*I, *shi*B, *ter*C, *yeh*A, *yeh*B, *yeh*C, *yeh*D

#### Diverse ARGs with sequence-type dependent distribution in ESBL-producing *E. coli*

Among the 20 ESBL-producing *E. coli* isolates sequenced, 34 different ARGs conferring resistance to 12 antimicrobial classes were found, ranging from 7 to 15 genes per isolate (Table 5). Isolates from experiment 1 had a more diverse ARGs with five *bla* genes detected including the CTX-M types. On the other hand, all the isolates from composting 2 carried only *bla*_CTX-M-55_. Regardless of ST or experiment, all isolates carried one or more genes that confer resistance to five antimicrobial classes: aminoglycosides, beta-lactams, sulfonamides, phenicols, and tetracyclines. Additional ARGs identified via WGS that were otherwise not included in antimicrobial susceptibility testing were *glp*T and *uhp*T*, arr*-2, *nfs*A, and *pmr*B which confer resistance to fosfomycin, rifamycin, nitrofuran, and polymyxins, respectively. The presence of ARGs within each ST stratified by antimicrobial class is shown in Table S5. Fosfomycin resistance genes were more widespread occurring in 15 isolates: *glp*T was detected by itself in all 10 isolates from experiment 2 and from two ST189 isolates from experiment 1; three isolates carried both *glp*T and *uhp*T genes. On the other hand, rifamycin resistance gene was detected only in two isolates belonging to ST189 in experiment 1; nitrofuran and polymyxin resistance genes were exclusively found in the isolates obtained from experiment 2 (Table 5; Table S5).

Phenotypic and genotypic associations of the isolates by their MIC values are presented in Table S6. This genotypic resistance profile also corresponds to phenotypic susceptibility profile, i.e., phenotypic resistance has at least one corresponding resistance gene. In addition, the two ESBL-producing *E. coli* from experiment 1 which were phenotypically susceptible to the two fluroquinolone drugs ciprofloxacin (MIC ≤ 0.015) and nalidixic acid (MIC = 2) were also negative for any gene conferring resistance to quinolones. On the other hand, there were some isolates that were classified as susceptible according to CLSI MIC breakpoints but carried ARGs to two critically important priority antibiotics. Macrolide resistance gene, *mph*(A), was detected in three isolates with MIC of 16. Isolates with MIC of 4 or 8 did not have any macrolide resistance gene detected. Two isolates with azithromycin resistance phenotype (MIC ≥ 32) belonging to ST189 had both macrolide [*mph*(A)] and lincosamide [*lnu*(F)] resistance genes suggesting that their combined presence gives higher level macrolide-lincosamide resistance. Similarly, isolates from experiment 1 which were phenotypically classified as susceptible or intermediate to ciprofloxacin (MIC 0.12–0.5) and susceptible to nalidixic acid (MIC 4–16), and one isolate phenotypically resistant to ciprofloxacin (MIC = 1) but susceptible to nalidixic acid (MIC = 8) had plasmid-mediated quinolone resistance genes *qnr*S1 and *qnr*B19. On the other hand, all isolates from the second experiment had ciprofloxacin MIC > 4 and nalidixic acid >64 contained chromosomally mediated quinolone resistance genes (*gyr*A, *par*C, and *par*E) suggesting that chromosomally mediated quinolone resistance genes confer high-level resistance.

Three aminoglycoside resistance gene groups were identified: aminoglycoside O-phosphotransferase gene (*aph*) that confers resistance to kanamycin and streptomycin, ANT(3”)-Ia family aminoglycoside nucleotidyltransferase gene (*aad*) conferring resistance to streptomycin, and aminoglycoside N-acetyltransferase gene (*aac*) that confers resistance to gentamicin. All isolates were highly resistant to tetracycline (MIC ≥ 32) and carried *tet*(A); in addition, two isolates (ST515) which were resistant to five antimicrobial classes (str-amp-axo-sul-chl-tet phenotype) carried *tet*(M), the more MDR isolates (ST189) that were additionally resistant to trimethoprim-azithromycin-ciprofloxacin carried *tet*(B). The two sulfonamide resistance genes differentially occurred such that *sul*2 was detected in isolates that were resistant to streptomycin, while *sul*3 was found in isolates that were resistant to both gentamicin and streptomycin also carrying the associated aminoglycoside resistance genes (Table S6).

#### Sequence type-dependent plasmid profiles among ESBL-producing *E. coli* isolates

Plasmids were found in all 20 ESBL-producing *E. coli* isolates (Table 5). Six plasmid types—IncF, IncH, IncR, Col, IncN, and p0111—with 10 replicons were detected. While all 10 isolates from experiment 2 (i.e., ST2509) and two ST10 isolates had only one plasmid, ST515 isolates had four plasmids. The plasmid profile also differs by ST, with isolates within ST10 being grouped into two categories with three isolates having three plasmids and the remaining two had only one plasmid.

#### Diversity of virulence factor genes within and between sequence types of ESBL-producing *E. coli*

The functions of the VFGs detected in this study are shown in Table S7. Across all 20 isolates, 30 VFG accessions were found that aligned to 29 VFG types (7–21 genes per isolate). Three VFGs*—hly*E, *nlp*I, and *ter*C—occurred in all isolates and two other VFGs*—csg*A and *gad*—were absent only from an isolate with ST6416. Isolates within each ST had similar VFG profiles except two isolates in ST10 which had their own unique profile that was different from the remaining three isolates (Table 5). While *asl*A was found in all isolates from experiment 1, and not in any of the isolates from experiment 2, fimbriae subunit proteins (F17A, F17D, and F17G), *lpf*A and *shi*B were found only in all isolates from experiment 2, and not in any isolate from experiment 1. Isolates in ST10 had the most diverse VFGs detected and were grouped into three categories with respect to their VFG distribution. Isolates in the other STs had similar VFG profiles.

### Variation of enterococcal species diversity both by experiment and composting stage

Species composition of generic and TET^r^ *Enterococcus* isolates is presented in Table 6. Generic enterococcal isolates obtained from dairy manure and manure mix samples collected from experiment 1 were almost exclusively composed of *E. faecium*, with only one *E. durans* isolate. The generic enterococcal isolates from manure mix and dairy manure samples collected during experiment 2 consisted of two spp., while *E. faecium* constituted the majority of the isolates (63.2%), *E. durans* constituted 36.8%. However, the final compost had a more diverse generic *Enterococcus* species, seven species from experiment 1 and four species from experiment 2. While enterococcal species either persisted (*durans* in experiment 1, and one *faecium* isolate from both experiments) or emerged (*faecalis*, *casseliflavus*, *hirae*, and *mundtii*), *E. faecium* isolates were almost eliminated during composting of the dairy manure.

**TABLE 6:** Generic and tetracycline-resistant (TET^r^) enterococcal species identified from composting of dairy manure amended with increasing levels of biochar

Bacterial type	*Enterococcus* spp.	Experiment 1			Experiment 2			Total
		Dairy manure	Manure mix	Final compost	Dairy manure	Manure mix	Final compost	
Generic enterococci	*E. casseliflavus*	0	0	1	0	0	2	3
	*E. durans*	1	0	4	0	7	0	12
	*E. faecalis*	0	0	3	0	0	4	7
	*E. faecium*	15	16	1	1	12	1	46
	*E. hirae*	0	0	3	0	0	0	3
	*E. mundtii*	0	0	3	0	0	1	4
	*E. species*	0	0	1	0	0	0	1
	**Total**	**16**	**16**	**16**	**1**	**19**	**8**	**76**
TET^r^ enterococci	*E. casseliflavus*	0	0	4	0	0	2	6
	*E. durans*	0	2	1	0	1	0	4
	*E. faecalis*	0	0	1	0	0	1	2
	*E. faecium*	13	11	2	0	2	2	30
	*E. hirae*	3	3	0	1	17	0	24
	**Total**	**16**	**16**	**8**	**1**	**20**	**5**	**66**

TET^r^ *Enterococcus* isolates obtained from dairy manure and initial manure mix collected in experiment 1 were composed primarily of *E. faecium* (Table 6). In experiment 2, *E. hirae* was the predominant species identified from the dairy manure and initial mix samples. TET^r^ *Enterococcus* isolates obtained from the final compost consisted of a more diverse species. While *E. hirae* were completely removed, and *E. faecium* and *E. durans* significantly reduced, *E. faecalis* and *E. casseliflavus* species emerged during the composting process.

### Phenotypic and genotypic resistance of the *Enterococcus* isolates

Antimicrobial resistance of generic and TET^r^ *Enterococcus* isolates is presented in Table 7. Quinupristin-dalfopristin resistance was the most prevalent among both generic and TET^r^ *Enterococcus* isolates. Antimicrobial resistance profile of both bacterial types is presented in Table 8. All generic *Enterococcus* isolates were susceptible to seven of the antimicrobials (ampicillin, avilamycin, chloramphenicol, gentamicin, linezolid, streptomycin, and vancomycin) regardless of sample type and experiment. For six antimicrobials, their level of resistance and distribution by sample type were similar between the two experiments. While tigecycline resistance (occurred only in experiment 1) was eliminated by composting, resistance against daptomycin, nitrofurantoin, quinupristin-dalfopristin, and tetracycline persisted the composting process. On the other hand, ciprofloxacin and erythromycin-resistant populations emerged in the final compost. Only 9.2% of generic *Enterococcus* isolates (*n* = 76) were MDR by being resistant to 3 (7.9%; two isolates from fresh manure, one isolate from the initial mix, and three isolates from final compost) or 4 (1.3%; one isolate from compost sample) antimicrobial classes. The remaining isolates were pan-susceptible (5.3%) or resistant to drugs in one (48.7%) or two (36.8%) antimicrobial classes. The single resistance was dominated by quinupristin-dalfopristin resistance.

**TABLE 7:** Antimicrobial resistance of generic *Enterococcus* and tetracycline-resistant (TET^r^) *Enterococcus* isolates obtained from dairy manure, mix, and compost^*b*^

Bacterial type	Antimicrobial agent	Experiment 1			Experiment 2		
		Dairy manure (*n* = 16)	Manure mix (*n* = 16)	Final compost (*n* = 16)	Dairy manure (*n* = 1)	Manure mix (*n* = 19)	Final compost (*n* = 8)
Generic enterococci	Ciprofloxacin	0	0	6.3	0	0	12.5
	Daptomycin	0	6.3	6.3	0	5.3	12.5
	Erythromycin	0	0	12.5	0	0	12.5
	Nitrofurantoin	6.3	18.8	6.3	0	26.3	12.5
	Quinupristin-dalfopristin^*a*^	81.3	93.8	69.2 (*n* = 13)	100	85.7	100 (*n* = 4)
	Tetracycline	18.8	6.3	43.4	0	15.8	25.0
	Tigecycline	68.8	12.5	0	0	0	0

		Dairy manure (*n* = 15)	Manure mix (*n* = 16)	Final compost (*n* = 6)	Dairy manure (*n* = 1)	Manure mix (*n* = 15)	Final compost (*n* = 4)
TET^r^ enterococci	Ampicillin	0	0	0	0	6.7	0
	Avilamycin	0	6.3	0	0	0	0
	Ciprofloxacin	6.7	6.3	33.3	0	0	0
	Erythromycin	6.7	0	50.0	0	0	50.0
	Gentamicin	0	0	0	0	0	25.0
	Nitrofurantoin	0	12.5	0	0	6.7	0
	Streptomycin	0	0	0	0	0	25.0
	Quinupristin-dalfopristin^*a*^	93.3	81.3	100 (*n* = 5)	100	73.3	100 (*n* = 3)
	Tetracycline	100	100	100	100	100	100
	Tigecycline	0	0	0	0	6.7	0

^*a*^ Since *E. faecalis* (*n* = 4 from final compost for generic enterococci from experiment 2; one isolate from TET^r^ from final compost) is intrinsically resistant to quinupristin/dalfopristin, the isolates were excluded from the prevalence calculation.

^*b*^ Only antimicrobial agents with resistant isolates are shown. All generic *Enterococcus* isolates were susceptible to ampicillin, avilamycin, chloramphenicol, gentamicin, linezolid, streptomycin, and vancomycin. All TET^r^ *Enterococcus* isolates were susceptible to chloramphenicol, daptomycin, linezolid, and vancomycin.

**TABLE 8:** Antimicrobial resistance profiles of generic and tetracycline (TET^r^) *Enterococcus* species^*a*^

Bacterial type	Experiment 1				Experiment 2			
	Sample type	*Enterococcus* species	Profile	No.	Sample type	*Enterococcus* species	Profile	No.
Generic enterococci	Dairy manure (*n* = 16)	*E. durans* (*n* = 1)	syn_tet_tig_	1	Dairy manure (*n* = 1)	*E. faecium* (*n* = 1)	syn_	1
		*E. faecium* (*n* = 15)	nit_syn_	1				
			pan susceptible	2				
			syn_	2				
			syn_tet_tig_	1				
			syn_tig_	8				
			tet_tig_	1				
	Initial mix (*n* = 16)	*E. faecium* (*n* = 16)	dap_syn_	1	Initial mix (*n* = 19)	*E. durans* (*n* = 7)	nit_syn_	2
			nit_syn_	2			pan susceptible	1
			nit_tet_	1			syn_	1
			syn_	10			syn_tet_	3
			syn_tig_	2		*E. faecium* (*n* = 12)	dap_nit_syn_	1
							nit_syn_	2
							pan susceptible	1
							syn	8
	Final compost (*n* = 16)	*E. casseliflavus* (*n* = 1)	syn_	1	Final compost (*n* = 8)	*E. casseliflavus* (*n* = 2)	cip_syn_	1
		*E. durans* (*n* = 4)	nit_	1			syn_	1
			syn_	1		*E. faecalis* (*n* = 4)	ery_syn_tet_	1
			syn_tet_	2			syn_	3
		*E. faecalis* (*n* = 3)	ery_syn_tet_	2		*E. faecium* (*n* = 1)	dap_nit_syn_tet_	1
			syn_	1		*E. mundtii* (*n* = 1)	syn_	1
		*E. faecium* (*n* = 1)	cip_syn_	1				
		*E. hirae* (*n* = 3)	dap_tet_	1				
			tet_	2				
		*E. mundtii* (*n* = 3)	syn_	3				
		*Enterococcus* spp. (*n* = 1)	syn_	1				
TET^r^ enterococci	Dairy manure (*n* = 15)	*E. faecium* (*n* = 12)	cip_syn_tet_	1	Dairy manure (*n* = 1)	*E. hirae* (*n* = 1)	syn_tet_	1
			ery_tet_	1				
			syn_tet_	10				
		*E. hirae* (*n* = 3)	syn_tet_	3				
	Initial mix (*n* = 16)	*E. durans* (*n* = 2)	syn_tet_	2	Initial mix (*n* = 15)			
		*E. faecium* (*n* = 11)	avi_avi_cip_nit_tet_	1		*E. faecium* (*n* = 2)	syn_tet__	2
			nit_syn_tet_	1		*E. hirae* (*n* = 12)	amp_syn_tet_	1
			syn_tet_	8			syn_tet_	7
			tet_	1			tet_	4
		*E. hirae* (*n* = 3)	syn_tet	2		*E. durans* (*n* = 1)	nit_syn_tet_tig_	1
			tet_	1				
	Final compost (*n* = 6)	*E. casseliflavus* (*n* = 3)	cip_ery_syn_tet_	1	Final compost (*n* = 4)	*E. casseliflavus* (*n* = 2)	ery_syn_tet_	1
			ery_syn_tet_	1			str_syn_tet_	1
			syn_tet_	1		*E. faecalis* (*n* = 1)	syn_tet_	1
		*E. durans* (*n* = 1)	syn_tet_	1		*E. faecium* (*n* = 1)	ery_gen_syn_tet	1
		*E. faecalis* (*n* = 1)	ery_syn_tet_	1				
		*E. faecium* (*n* = 1)	cip_syn_tet_	1				

^*a*^ Drug abbreviations: amp, ampicillin; avi, avilamycin; cip, ciprofloxacin; dap, daptomycin; ery, erythromycin; gen, gentamicin; nit, nitrofurantoin; str, streptomycin; syn, quinupristin-dalfopristin; tet, tetracycline; tig, tigecycline.

Among TET^r^ *Enterococcus* isolates (*n* = 57), 21.1% were MDR by being resistant to drugs in three (14%; five isolates from compost, two from initial mix, and one isolate from fresh manure) or four (7%; two isolates each from initial mix and compost) antimicrobial classes. The remaining isolates were resistant only to tetracycline (10.5%) or other drugs in one additional antimicrobial class (68.4%). All TET^r^ *Enterococcus* isolates were susceptible to four antimicrobial drugs: chloramphenicol, daptomycin, linezolid, and vancomycin. Genotypic resistance among phenotypically tetracycline-resistant generic *Enterococcus* isolates (*n* = 16) was mainly (87.5%) conferred by *tet*(M). The remaining two isolates carried *tet*(L), which was detected from *E. hirae* obtained from final compost in experiment 1, or *tet*(P), which was detected from an *E. faecium* isolate obtained from final compost in experiment 2. Similarly, resistance among the TET^r^ isolates (*n* = 57) was mainly (93%) conferred by *tet*(M). The *tet*(P) gene was detected from three *E. casseliflavus* isolates from final compost (two from experiment 1 and one from experiment 2) with MDR profile. *tet*(L) was detected from one tetracycline only resistant *E. hirae* isolate from the initial mix in experiment 2.

## DISCUSSION

The two field-based composting experiments demonstrated clear and biologically meaningful differences in how individual components—biochar, sawdust, raw manure, and the composting mixture—contributed to the antimicrobial-resistant bacterial pool. The results underscore that raw dairy manure is the principal and overwhelming source of both generic and antimicrobial-resistant bacteria in the composting system because neither biochar nor sawdust introduced clinically important resistance phenotypes into the system. The finding that biochar was essentially bacteriologically sterile confirms that pyrolysis-derived materials (typically produced at 300°C to 700°C) pose minimal microbiological risk when incorporated into manure management systems (9). Even the single enrichment-positive sample suggests accidental contamination rather than biological carryover. While biochar did not exert a statistically measurable antimicrobial effect, this absence is itself meaningful: the lack of added benefit indicates that composting’s inherent microbial and thermal dynamics already drive bacterial reductions to near completion, making additive effects difficult to detect. The slightly more pronounced reductions at the 10% biochar level hint at a possible physicochemical facilitation mechanism—such as moisture regulation or improved aeration—but the effect size is too small for firm conclusions. This suggests future work should test higher replication or focus on mechanistic interactions between biochar surface chemistry and compost microbiota.

Bulking agents such as sawdust are commonly added to animal manure to increase oxygen penetration, for proper moisture, and to maintain C:N ratio to support optimum microbial growth during composting (7, 8). However, there are concerns that bulking agents can be a source of bacteria. Although generic *E. coli*, TET^r^ *E. coli*, generic enterococci, and TET^r^ enterococci were detected from 94% to 100% of the sawdust samples after enrichment, they were found at low concentrations in the sawdust samples. Interestingly, sawdust was negative for 3GC^r^ *E. coli* and ESBL-producing *E. coli* indicating that sawdust does not provide bacteria resistant to 3rd and higher generation cephalosporins that are critically important and priority antibiotics for human use. Consistent with our findings, samples of rye silage used as a bulking agent only had generic *Enterococcus* concentrations above the detection limit with no detectable antibiotic-resistant bacteria or generic *E. coli* concentrations during the summer-fall (August–September) composting of beef cattle manure (8). Our study clearly showed that sawdust, although positive for several bacterial groups after enrichment, consistently exhibited low bacterial concentrations and an absence of 3GC^r^ and ESBL-producing *E. coli*. This distinguishes sawdust as a low-risk-amplifying bulking agent. The finding confirms that environmental or plant-derived bulking materials do not serve as reservoirs of high-priority antimicrobial-resistant bacteria. Therefore, any resistant populations observed during composting can be attributed mainly to manure inputs and composting dynamics, not cross-contamination from bulking amendments.

Across sample types, composting itself—rather than biochar—emerged as the primary driver of reductions in both bacterial concentration and prevalence in dairy cattle manure. The mechanistic basis is likely multifactorial, involving sustained thermophilic phases, microbial competition, moisture reduction, and depletion of available nutrients (21–24). The first effect was observed in the form of a significant reduction in the concentrations and prevalence of generic *E. coli*, TET^r^ *E. coli*, generic enterococci, and TET^r^ enterococci from their 100% prevalence in fresh dairy manure. The second effect was its complete removal of 3GC^r^ *E. coli* and ESBL-producing *E. coli*. The complete elimination of 3GC^r^ and ESBL-producing *E. coli* and the substantial reductions of TET^r^ *E. coli* demonstrate that composting environments can disrupt the survival of Gram-negative bacteria, even those with multidrug-resistant profiles. This is especially important given that these organisms carried bla_CTX-M_ and other clinically relevant AMR determinants. The data indicate that composting can interrupt environmental dissemination pathways for pathogens considered critical by WHO and CDC, making it a valuable pre-harvest mitigation tool.

However, composting effectiveness was greatly influenced by the composting season reflected in the setup of the two experiments. Composting during spring-summer (experiment 2) greatly reduced the prevalence of generic enterococci and completely removed generic and TET^r^ *E. coli*. Thus, this leads to a conclusion that composting has a differential impact on Gram-positive and Gram-negative bacteria. While Gram-negative bacteria, such as *E. coli* including those resistant to antibiotics, are readily eliminated by composting, Gram-positive bacteria such as enterococci, including antibiotic-resistant ones, can survive composting. Although similar seasonal effects of composting have been inferred previously (8), reporting of the differential impact based on bacterial type is novel. The seasonal differences between experiments provide an additional analytical layer. Spring–summer composting resulted in more complete reductions of generic and TET^r^ *E. coli*, supporting the interpretation that environmental heat and extended thermophilic durations synergize with biological degradation processes. These findings emphasize that composting efficacy is not uniform across seasons and that managing compost conditions to mimic warmer-season dynamics (e.g., controlled aeration, optimized moisture) may produce more consistent resistance-reduction outcomes.

Another notable finding was the differential effect of composting on *Enterococcus* species. In contrast to *E. coli*, enterococci showed substantial resilience, remaining detectable after composting and diversifying in species composition. This differential survival suggests that Gram-positive organisms—owing to thicker peptidoglycan layers, stress-resistant physiology, and possible biofilm formation—are less affected by composting conditions. Of the two *Enterococcus* species of public health importance, while *E. faecium* was significantly reduced by composting, *E. faecalis* repopulated in addition to a more environmental species such as *E. casseliflavus*, *E. durans*, *E. mundtii*, and *E. hirae*. The emergence of these *Enterococcus* species in final compost samples indicates ecological succession rather than mere survival. This finding suggests that composting not only eliminates certain populations but also selects for species adapted to environmental conditions, implying that enterococci could serve as robust indicators of compost process performance. Because some enterococcal species carry clinically significant resistance traits, their persistence highlights a residual risk pathway that warrants monitoring. *Enterococcus* species frequently contaminate fresh produce, a significant proportion of these isolates, especially *E. faecium*, are resistant to clinically important antibiotics and often multidrug-resistant. Irrigation and recycled wastewater, fresh animal manure and inadequately composted animal manure can serve as important routes for contamination. While no outbreaks have been directly linked to vegetable-derived *Enterococcus*, the presence of resistant strains in consumed produce raises concerns for gastrointestinal colonization, potential spread of resistance genes, and opportunistic infections (25, 26). Our findings alongside these studies suggest the need for a thorough composting of animal manure using Gram-positive fecal bacteria such as enterococci as performance indicators since these were found to survive our current dairy manure composting study.

On the other hand, composting effectively removed both generic *E. coli* in experiment 2 and isolates resistant to antibiotics considered highly important (TET^r^ *E. coli*) or highest priority critically important (3GC^r^ and ESBL *E. coli*) antibiotics according to World Health Organization ranking of antimicrobials (27, 28). It is worth mentioning notable features of the resistant populations obtained from fresh dairy manure. First, TET resistance co-selects only the older antimicrobials (ampicillin, chloramphenicol, streptomycin, and sulfisoxazole) to a lesser degree or occurs by itself, it did not co-select for resistance to most critically important antibiotics (gentamicin, quinolones, and most importantly ceftriaxone which was used for the isolation of 3GC^r^ *E. coli* and ESBL-producing *E. coli* isolates). On the contrary, tetracycline resistance is readily co-selected with the 3GC^r^ *E. coli* and ESBL-producing *E. coli*. This finding is consistent with our previous observations in different settings which have significant implications in the spread and persistence of antimicrobial-resistant *E. coli* in the environment (2, 13).

Second, MDR is the hallmark of the 3GC^r^ *E. coli* and ESBL-producing *E. coli* isolates obtained from fresh dairy manure which is also consistent with other studies (2, 13). However, the peculiarity in this study was that the antibiotic-resistant isolates (TET^r^ *E. coli*, 3GC^r^ *E. coli*, and ESBL-producing *E. coli*) represented two distinct populations between the two experiments. Isolates from experiment 1 were more diverse with respect to resistance profiles, belonging to four STs, antimicrobial resistance genes, plasmid profiles, and virulence factor genes. On the other hand, isolates from experiment 2 were clonal with respect to these features. Although factors that drive for this variation in the same dairy farm need further study, it is plausible that while the resistance and virulence factors from experiment 1 disseminate through horizontal transfer, they are chromosomally mediated in experiment 2 and disseminated through bacterial transmission from animal to animal within the dairy herd. This was evident in the distribution of the quinolone resistance genes: while isolates from experiment 1 carried plasmid-mediated genes (*qnr*B, *qnr*S), all isolates from experiment 2 carried the chromosomally mediated genes *gyr*A, *par*C, and *par*E. The distinct AMR profiles observed between experiments—diverse and horizontally acquired resistance in experiment 1 versus clonal, chromosomally mediated resistance in experiment 2—suggest that the source herd harbored two different dominant AMR populations across seasons. This highlights a critical insight: manure AMR characteristics are dynamic and can shift based on farm-level antimicrobial usage patterns or animal-to-animal transmission cycles. Therefore, composting systems should be evaluated over multiple seasons to capture these fluctuating risk profiles.

All isolates, whether they were isolated by media supplemented with cefotaxime or obtained on a commercially available ESBL plates, carried the *bla*_CTX-M-15_ group as genotypically confirmed both by PCR and WGS of subpopulations to be ESBL-producing *E. coli*. Extended spectrum beta-lactamases confer resistance to a broader range of beta-lactams (13) and, as also shown in the present study, are often associated with MDR (29–31). ESBL *E. coli* has been reported from dairy cows (32–34). Although studies are needed to identify modifiable risk factors to understand the dynamics of ESBL *E. coli* within dairy farms over a full lactation cycle, antimicrobial uses can be a primary factor. Antimicrobials are required on dairy cattle farms for the treatment and prevention of bacterial infections particularly mastitis (10). Beta-lactams including penicillin and cephalosporins are the major antimicrobials used on dairy farms (10). On the dairy farm from which the manure was obtained, beta-lactams were the main antibiotics used for the treatment of mastitis (cephapirin—1st generation cephalosporin, ceftiofur—3rd generation cephalosporin) and generalized infections (ampicillin) and for the prevention of mastitis in the form of dry cow therapy (cephapirin, cephapirin benzathine, penicillin and dihydrostreptomycin, cloxacillin benzathine). Other antibiotics used were florfenicol (amphenicol class), tulathromycin (a macrolide), and oxytetracycline (tetracycline). Nevertheless, regardless of experimental setup (whether in spring/summer or fall/winter) or biochar amendment, composting effectively removed the ESBL-producing *E. coli* from dairy manure. This study provides strong evidence that composting, a simple and cost-effective animal manure management, can effectively eliminate MDR bacteria which are classified as critical priority pathogens by WHO (35) or serious threat to public health by CDC (36) from the dairy manure limiting their dissemination beyond farm for a safer utilization of dairy manure.

In conclusion, biochar is a bacteriologically sterile substance with no added effect on the prevalence and concentrations of bacteria and their antimicrobial-resistant populations during composting of dairy manure. However, further research, preferably a multicenter study, is needed with multiple composting experiments using 10% biochar which showed limited impact. Given its unique characteristics as free of bacteria, a rich carbon source, and its porosity, biochar can be evaluated as a bulking agent for manure composting. The presence of aminopyralid herbicide residue in the dairy manure does not affect composting process in reducing generic and antibiotic-resistant bacteria. Fresh dairy manure contained high prevalence and concentrations of generic and resistant bacterial populations of significant public health importance. However, while composting effectively removed *E. coli* including antibiotic-resistant populations, it was not effective in removing the *Enterococcus* populations. Therefore, this finding indicates that enterococci should be considered performance indicators for animal manure composting. Nevertheless, composting effectively eliminated ESBL *E. coli* considered critical priority pathogens from the dairy manure limiting their dissemination beyond farm for a safer utilization of dairy manure. In general, the elimination of MDR *E. coli* but persistence of enterococci demonstrates that composting selectively reduces the organisms of highest public health concern while leaving behind bacteria of lower clinical significance. This selectivity strengthens the argument for composting as a practical antimicrobial-resistance mitigation tool. However, it also underscores the need for composting standards that ensure sufficient time and temperature thresholds to reduce resistant Gram-positive populations.

## Data Availability

Whole-genome sequences of 20 isolates (10 3GC^r^ *E. coli* and 10 ESBL-producing *E. coli*) obtained from dairy cattle manure for the two field composting experiments were submitted to the NCBI Sequence Read Archive under BioProject no. PRJNA1146066. Individual sequences can be obtained with accession numbers ranging from SAMN62049898 to SAMN62049917 and can be accessed at https://www.ncbi.nlm.nih.gov/sra/PRJNA1146066. All supporting data are provided within the article and its supplemental material.

## SUPPLEMENTAL MATERIAL

Supplemental material aem.01258-26-s0001.pdf Supplemental text; Tables S1 to S7.

ASM does not own the copyrights to Supplemental Material that may be linked to, or accessed through, an article. The authors have granted ASM a non-exclusive, world-wide license to publish the Supplemental Material files. Please contact the corresponding author directly for reuse.
