# Supplementary material for: Composting dairy cattle manure significantly reduces antibiotic-resistant bacteria with no biochar impact
Source: Appl Environ Microbiol. 2026 Aug 13;92(9):e01258-26. doi: 10.1128/aem.01258-26 (PMC13599537; doi:10.1128/aem.01258-26)
Supplement: Supplementary file 1 [file aem.01258-26-s0001.pdf]

**SUPPLEMENTAL MATERIALS**

for

# Composting dairy cattle manure significantly reduces antibiotic resistant bacteria with no biochar impact

Getahun E. Agga[1]*, Annesly Netthisinghe[2], Paul Woosley[2], William Strunk[2], Karamat R. Sistani[1]

[1] Food Animal Environmental Systems Research Unit, Agricultural Research Service, U.S. Department of Agriculture, Bowling Green, KY, USA; [2] Department of Agriculture and Food Science, Western Kentucky University, Bowling Green, KY, USA.

*Corresponding author

email: Getahun.agga@usda.gov

## Supplementary Text

**Biochar and sawdust addition to pre-composting of dairy manure do not significantly increase its bacterial load.** Experiment 1 was used to assess the prevalence and concentrations of generic and antimicrobial resistant *E. coli* and enterococci in individual components (biochar, sawdust, and raw manure) before they were mixed and distributed to the composting reactors. the Overall prevalence of the targeted bacteria by sample type is shown in Figure 2. Only a single biochar sample was prevalence positive (i.e. after secondary enrichment) both for the generic *E. coli* and enterococci. The antibiotic-resistant bacteria ($TET^r$ *E. coli,* $3GC^r$ *E. coli,* ESBL-producing *E. coli*, and $TET^r$ enterococci) were neither enumerable nor detectable from any of the biochar samples (n=12).

All sawdust samples (n=16) were prevalence positive for generic *E. coli*, TET$^r$ *E. coli*, and generic enterococci following secondary enrichments, and 93.8% (15/16) of them were positive for TET$^r$ enterococci. 3GC$^r$ *E. coli* and ESBL-producing *E. coli* were not detected from any of the sawdust samples. On the other hand, all manure samples (n=17) were prevalence positive for generic *E. coli*, TET$^r$ *E. coli*, 3GC$^r$ *E. coli*, ESBL-producing *E. coli*, generic enterococci, and TET$^r$ enterococci after secondary enrichment.

Marginal predicted mean concentrations of generic, and tetracycline resistant *E. coli* and enterococci isolates are shown in Figure 3. Concentrations of generic *E. coli* ($P$= 0.9262), TET$^r$ *E. coli* ($P$= 0.7529), generic enterococci ($P$= 0.5399), and TET$^r$ enterococci ($P$= 0.7188) in the individual components did not significantly differ by %BC, thus data were combined for comparative analysis. Sample type had a significant effect ($P$ <0.0001) on the concentrations of generic *E. coli*, TET$^r$ *E. coli*, generic enterococci, and TET$^r$ enterococci. Sawdust had the lowest concentrations ($P$ <0.001) of these bacteria compared to raw manure or manure mixture. Generic *E. coli* and generic enterococci were enumerable from six, and seven sawdust samples with the mean concentrations of 1.7 and 2.0 $log_{10}$ CFUs/g, respectively. TET$^r$ *E. coli* and TET$^r$ enterococci were enumerable at concentrations of 1.3 $log_{10}$ CFUs/g only from a single same sawdust sample.

Mean generic *E. coli* concentration of 4.6 $log_{10}$ CFUs/g in the manure did not significantly differ ($P$=0.117) from that of the manure mix. However, the mean TET$^r$ *E. coli* concentration (4.0 $log_{10}$ CFUs/g) in the manure was significantly higher ($P$ = 0.021) than that of the manure mix. 3GC$^r$ *E. coli* were enumerated from eight manure samples yielding a mean concentration of 1.5 $log_{10}$ CFUs/g; however, they were not detected from any manure mix samples in experiment 1. ESBL-producing *E. coli* were not enumerable from any of the dairy manure samples. Generic

enterococci ($P$=0.166) and TET$^r$ enterococci ($P$=1.00) were enumerable from all manure samples, and their mean concentrations of 4.4 and 3.3 $log_{10}$ CFUs/g, respectively did not statistically differ from manure mix samples.

**Effect of biochar amended dairy manure composting on concentrations and prevalence of generic and antibiotic-resistant bacteria.** Experiment 2 was used to evaluate the effect of aminopyralid herbicide on the prevalence and concentrations of generic and antibiotic-resistant *E. coli* and enterococci. Aminopyralid did not have any significant effect on the mean concentrations of generic *E. coli* ($P$= 0.1294), TET$^r$ *E. coli* ($P$= 0.0723), generic enterococci ($P$= 0.3702), and TET$^r$ enterococci ($P$= 0.7267) in manure mix. 3GC$^r$ *E. coli* were enumerable from four manure mix samples (2 per aminopyralid treatment) each with a concentration of 1.3 $log_{10}$ CFUs/ml. Generic enterococci concentration in the compost samples was not significantly ($P$= 0.8669) affected by aminopyralid addition. TET$^r$ enterococci was enumerable only from a single compost sample without aminopyralid with a concentration of 1.8 $log_{10}$ CFUs/g. Generic *E. coli*, TET$^r$ *E. coli*, 3GC$^r$ *E. coli,* and ESBLs *E. coli* were not enumerable (nor detectable) from the compost samples in experiment 2, regardless of aminopyralid treatment. Thus, concentration data were combined by sample type to statistically analyze the effects of composting with or without biochar amendment.

The mean concentrations of generic and tetracycline resistant *E. coli* and enterococci isolates in the manure mix and compost by biochar percentage is shown Table 2. All manure mixture samples (n=36; 9 samples per %BC) were positive by enumeration for generic *E. coli*, TET$^r$ *E. coli* (except one sample from the 4%BC in Experiment 2), generic enterococci, and TET$^r$ enterococci. Their concentrations in the manure mixture did not significantly differ between the two experiments ($P$ >0.05), thus enumeration data were combined by %BC for further analysis.

Composting significantly reduced the number of samples with enumerable bacterial concentrations, depending on the experiments and bacterial type. $TET^r$ *E. coli*, $3GC^r$ *E. coli*, and ESBL-producing *E. coli* were not enumerable from any compost sample in both experiments. All compost samples (n=20) in Experiment 2 were negative by enumeration for generic *E. coli*, and only eight compost samples from Experiment 1 (n=16; two of the 0% BC, two of the 2% BC, and four of the 4% BC) were positive by enumeration. Similarly, only 13 compost samples (8 in Experiment 1, and 5 in Experiment 2) were positive for generic enterococci by enumeration. $TET^r$ enterococci were enumerated only from two final compost samples both from 0% BC in both experiments, with a mean concentration of 2.0 $log_{10}$ CFUs/g.

Biochar amendment did not have a statistically significant effect on generic *E. coli* ($P$= 0.3523), generic enterococci ($P$= 0.5182), and $TET^r$ enterococci ($P$= 0.9579). However, the highest biochar concertation (10% BC) seemed to have an added effect beyond composting alone; compost samples from the 10% BC treatment in Experiment 1 were negative by enumeration for all targeted bacteria, except for three samples which were enumeration positive for generic enterococci. Composting significantly ($P$<0.0001) reduced the mean concentrations of generic enterococci and $TET^r$ enterococci when compared to manure mixture, with no significant ($P$= 0.6838) effect on the mean concentration of generic *E. coli*.

$3GC^r$ *E. coli* and ESBLs *E. coli* were not detected from any compost samples. Biochar amendment did not have a significant effect on the prevalence of generic *E. coli* (Fisher's exact $P$= 0.584), $TET^r$ *E. coli* (Fisher's exact $P$= 1.00), generic enterococci (Fisher's exact $P$= 0.833), and $TET^r$ enterococci (Fisher's exact $P$= 1.00) in the final compost. Significant experimental effects on prevalence were observed for generic *E. coli* (Fisher's exact $P$<0.001), $TET^r$ *E. coli* (Fisher's exact $P$= 0.004), and generic enterococci (Fisher's exact $P$= 0.002), but not for $TET^r$

enterococci (Fisher's exact $P$= 0.169) in the final compost. Composting significantly (Fisher's exact $P$ <0.001) reduced the prevalence of generic *E. coli* (100% prevalence in manure mixture vs. 30.6% in the final compost). However, the reduction in prevalence was primarily attributed to experiment 2. While composting significantly ($P$= 0.043) reduced the prevalence of generic *E. coli* from 100% (n=16) in the manure mixture to 68.8% (n=16) in the final compost samples in experiment 1, it completely removed them in experiment 2. Composting significantly (Fisher's exact $P$ <0.001) reduced the prevalence of TET$^r$ *E. coli* (100% [n=36] prevalence in manure mixture vs. 16.7% [n=36] in the final compost). Again, this reduction in prevalence was primarily attributed to experiment 2. While composting significantly ($P$< 0.001) reduced the prevalence of TET$^r$ *E. coli* in the final compost compared to manure mixture (37.5% [n=16] vs. 100% [n=16], respectively) in experiment 1, it completely removed them in experiment 2. Composting significantly (Fisher's exact $P$ =0.002) reduced the prevalence of generic enterococci (100% prevalence in manure mixture vs. 75% in the final compost). This reduction, however, was exclusively attributed to experiment 2 (100% [n=16] prevalence in experiment 1 vs. 55% [n=20] in experiment 2). TET$^r$ enterococci prevalence in the final compost did not significantly ($P$=0.169) differ between experiments (50% in experiment 1 vs 25% in experiment 2). Composting significantly ($P$<0.001) reduced the prevalence of TET$^r$ enterococci when comparing prevalence in the manure mix (100% [n=36]) versus the final compost (36.1% [n=36]).

**Table S1: Antimicrobial agents, concentration ranges, interpretive categories, and minimum inhibitory concentrations (MIC) breakpoints for tetracycline resistant-, 3rd generation cephalosporin resistant-, and extended spectrum beta-lactamase producing-*E. coli* isolates obtained from biochar amended dairy cattle manure composting**

| CLSI antimicrobial class | Antimicrobial agent | Concentration tested (µg/ml) | Interpretive categories and MIC breakpoints (µg/ml) | | |
|---|---|---|---|---|---|
| | | | S | I | R |
| Aminoglycosides | Gentamicin | 0.25-16 | ≤ 2 | 4 | ≥ 8 |
| | Streptomycin* | 2-64 | ≤ 16 | | ≥ 32 |
| Cephems | Cefoxitin | 0.5-32 | ≤ 8 | 16 | ≥ 32 |
| | Ceftriaxone | 0.25-64 | ≤ 1 | 2 | ≥ 4 |
| Macrolides | Azithromycin* | 0.25-32 | ≤ 16 | | ≥ 32 |
| Penems | Meropenem | 0.06-4 | ≤ 1 | 2 | ≥ 4 |
| Penicillins | Ampicillin | 1-32 | ≤ 8 | 16 | ≥ 32 |
| Quinolones | Ciprofloxacin | 0.015-4 | ≤ 0.25 | 0.5 | ≥ 1 |
| | Nalidixic acid | 0.5-32 | ≤ 16 | | ≥ 32 |
| β-lactam combination agents | Amoxicillin-clavulanic acid | 1/0.5-32/16 | ≤ 8/4 | 16/8 | ≥ 32/16 |
| Folate pathway antagonists | Sulfisoxazole | 16-256 | ≤ 256 | | ≥ 512 |
| | Trimethoprim-sulfamethoxazole | 0.12/2.38-4/76 | ≤ 2/38 | | ≥ 4/76 |
| Phenicols | Chloramphenicol | 2-32 | ≤ 8 | 16 | ≥ 32 |
| Tetracyclines | Tetracycline | 4-32 | ≤ 4 | 8 | ≥ 16 |
| Polymyxins | Colistin | 0.25-8 | ≤ 2 | | ≥ 4 |

*Had no Clinical & Laboratory Standards Institute (CLSI) breakpoint. The 2021 National Antimicrobial Resistance Monitoring System (NARMS) interpretive criteria for susceptibility testing were applied.

S=susceptible; I=intermediate; R=resistant

**Table S2: Antimicrobial agents, concentration ranges, interpretive categories, and minimum inhibitory concentrations (MIC) breakpoints for enterococcal isolates obtained from biochar amended dairy cattle manure composting**

| CLSI antimicrobial class | Antimicrobial agent | Concentration tested (µg/ml) | Interpretive categories and MIC breakpoints (µg/ml) | | |
|---|---|---|---|---|---|
| | | | S | I | R |
| Aminoglycosides | Gentamicin[a] | 16-1024 | ≤ 500 | | > 500 |
| | Streptomycin[a] | 64-2048 | ≤ 512 | | ≥ 1024 |
| Penicillins | Ampicillin | 0.25-32 | ≤8 | | ≥ 16 |
| Orthosomycin | Avilamycin[a] | 4-32 | | | ≥ 16 |
| Phenicols | Chloramphenicol | 2-64 | ≤ 8 | 16 | ≥ 32 |
| Quinolones | Ciprofloxacin | 0.12-16 | ≤ 1 | 2 | ≥ 4 |
| Lipopeptides | Daptomycin *E. faecium* only | 0.25-16 | ≤ 4 | | ≥ 8 |
| | Other enterococci spp. | | ≤ 2 | 4 | ≥ 8 |
| Macrolides | Erythromycin | 0.25-8 | ≤ 0.5 | 1–4 | ≥ 8 |
| Oxazolidinones | Linezolid | 0.5-8 | ≤ 2 | 4 | ≥ 8 |
| Nitrofurans | Nitrofurantoin | 2-512 | ≤ 32 | 64 | ≥ 128 |
| Streptogramins | Quinupristin-dalfopristin | 0.5-32 | ≤ 1 | 2 | ≥ 4 |
| Tetracyclines | Tetracycline | 1-32 | ≤ 4 | 8 | ≥ 16 |
| Glycylcycline | Tigecycline[a] | 0.015-0.5 | ≤ 0.25 | | ≥ 0.5 |
| Glycopeptides | Vancomycin | 0.25-32 | ≤4 | 8-16 | ≥ 32 |

[a] Had no Clinical & Laboratory Standards Institute (CLSI) breakpoint. The 2021 National Antimicrobial Resistance Monitoring System (NARMS) interpretive criteria for susceptibility testing were applied.

S=susceptible; I=intermediate; R=resistant.

**Table S3: Whole genome sequence read statistics of 20 third generation cephalosporin resistant (3GC) and extended spectrum β-lactamase (ESBL) producing *E. coli* obtained from dairy cattle manure.**

| | | Mean Read Score | | Q30 Passing (%) | | | |
|---|---|---|---|---|---|---|---|
| **Lab id** | NVSL Sample ID | **R1** | **R2** | **R1** | **R2** | **Sequence Depth** | **Genome Length** |
| WKUCMP1-E. coli-3GC-D0-CM-01 | 25G00437-001 | 39.08 | 38.58 | 98.50% | 97.20% | 214.4X | 5,000,000bp |
| WKUCMP1-E. coli-3GC-D0-CM-02 | 25G00437-002 | 39.12 | 38.34 | 98.60% | 96.50% | 192.4X | 5,000,000bp |
| WKUCMP1-E. coli-3GC-D0-CM-03 | 25G00437-003 | 39.18 | 38.23 | 98.90% | 96.50% | 222.4X | 5,000,000bp |
| WKUCMP1-E. coli-3GC-D0-CM-04 | 25G00437-004 | 39.1 | 38.38 | 98.80% | 96.80% | 166.8X | 5,000,000bp |
| WKUCMP1-E. coli-3GC-D0-CM-05 | 25G00437-005 | 39.06 | 38.39 | 98.60% | 97.00% | 161.4X | 5,000,000bp |
| WKUCMP1-E. coli-ESBL-D0-CM-01 | 25G00437-006 | 39.1 | 38.68 | 98.60% | 97.60% | 234.8X | 5,000,000bp |
| WKUCMP1-E. coli-ESBL-D0-CM-02 | 25G00437-007 | 39.05 | 38.54 | 98.40% | 97.20% | 204.4X | 5,000,000bp |
| WKUCMP1-E. coli-ESBL-D0-CM-03 | 25G00437-008 | 39.13 | 38.47 | 98.60% | 97.00% | 234.2X | 5,000,000bp |
| WKUCMP1-E. coli-ESBL-D0-CM-04 | 25G00437-009 | 39.05 | 38.51 | 98.60% | 97.20% | 187.2X | 5,000,000bp |
| WKUCMP1-E. coli-ESBL-D0-CM-05 | 25G00437-010 | 39.07 | 38.34 | 98.60% | 96.60% | 170.4X | 5,000,000bp |
| WKUCMP2-E. coli-3GC-D0-CM-01 | 25G00437-011 | 39.1 | 38.36 | 98.70% | 96.70% | 204.9X | 5,000,000bp |
| WKUCMP2-E. coli-3GC-D0-CM-02 | 25G00437-012 | 39.12 | 38.45 | 98.90% | 96.80% | 165.5X | 5,000,000bp |
| WKUCMP2-E. coli-3GC-D0-CM-03 | 25G00437-013 | 39.14 | 38.46 | 98.70% | 96.90% | 199.8X | 5,000,000bp |
| WKUCMP2-E. coli-3GC-D0-CM-05 | 25G00437-014 | 39.07 | 38.57 | 98.50% | 97.20% | 189.4X | 5,000,000bp |
| WKUCMP2-E. coli-3GC-D0-CM-06 | 25G00437-015 | 39.04 | 38.74 | 98.50% | 98.00% | 182.4X | 5,000,000bp |
| WKUCMP2-E. coli-ESBL-D0-CM-01 | 25G00437-016 | 39.12 | 38.5 | 98.70% | 97.10% | 228.0X | 5,000,000bp |
| WKUCMP2-E. coli-ESBL-D0-CM-02 | 25G00437-017 | 39.13 | 38.43 | 98.80% | 96.90% | 173.0X | 5,000,000bp |
| WKUCMP2-E. coli-ESBL-D0-CM-03 | 25G00437-018 | 39.11 | 38.35 | 98.70% | 96.60% | 177.8X | 5,000,000bp |
| WKUCMP2-E. coli-ESBL-D0-CM-05 | 25G00437-019 | 39.03 | 38.45 | 98.40% | 97.00% | 195.1X | 5,000,000bp |
| WKUCMP2-E. coli-ESBL-D0-CM-06 | 25G00437-020 | 39.15 | 38.46 | 99.00% | 97.10% | 171.6X | 5,000,000bp |

**Table S4: Whole genome sequence assembly statistics of 20 third generation cephalosporin resistant (3GC) and extended spectrum β-lactamase (ESBL) producing *E. coli* obtained from dairy cattle manure.**

| Lab id | NVSL Sample ID | Scaffolds | Total length | Longest scaffold | Scaffolds >1K nt | Genome >1K nt | N50 | L50 |
|---|---|---|---|---|---|---|---|---|
| WKUCMP1-E.coli-3GC-D0-CM-01 | 25G00437-001 | 231 | 4,837,179 | 395,316 | 82 | 99.16 | 165,812 | 10 |
| WKUCMP1-E.coli-3GC-D0-CM-02 | 25G00437-002 | 183 | 4,684,856 | 406,601 | 75 | 99.32 | 139,225 | 11 |
| WKUCMP1-E.coli-3GC-D0-CM-03 | 25G00437-003 | 259 | 4,847,159 | 395,316 | 79 | 98.96 | 169,865 | 10 |
| WKUCMP1-E.coli-3GC-D0-CM-04 | 25G00437-004 | 195 | 4,852,874 | 453,640 | 71 | 99.32 | 173,954 | 10 |
| WKUCMP1-E.coli-3GC-D0-CM-05 | 25G00437-005 | 223 | 4,964,907 | 592,942 | 82 | 99.24 | 135,377 | 9 |
| WKUCMP1-E.coli-ESBL-D0-CM-01 | 25G00437-006 | 248 | 4,814,188 | 453,925 | 72 | 99.04 | 204,597 | 9 |
| WKUCMP1-E.coli-ESBL-D0-CM-02 | 25G00437-007 | 294 | 4,978,989 | 592,746 | 89 | 98.93 | 135,377 | 10 |
| WKUCMP1-E.coli-ESBL-D0-CM-03 | 25G00437-008 | 210 | 4,815,122 | 352,266 | 77 | 99.28 | 133,428 | 11 |
| WKUCMP1-E.coli-ESBL-D0-CM-04 | 25G00437-009 | 193 | 4,810,033 | 352,266 | 75 | 99.39 | 141,630 | 10 |
| WKUCMP1-E.coli-ESBL-D0-CM-05 | 25G00437-010 | 184 | 4,796,381 | 453,630 | 71 | 99.38 | 198,038 | 9 |
| WKUCMP2-E.coli-3GC-D0-CM-01 | 25G00437-011 | 194 | 5,127,050 | 970,028 | 60 | 99.31 | 229,388 | 6 |
| WKUCMP2-E.coli-3GC-D0-CM-02 | 25G00437-012 | 154 | 5,117,705 | 970,028 | 60 | 99.5 | 275,254 | 5 |
| WKUCMP2-E.coli-3GC-D0-CM-03 | 25G00437-013 | 191 | 5,126,043 | 970,224 | 62 | 99.33 | 229,388 | 6 |
| WKUCMP2-E.coli-3GC-D0-CM-05 | 25G00437-014 | 160 | 5,118,619 | 970,028 | 59 | 99.49 | 249,400 | 5 |
| WKUCMP2-E.coli-3GC-D0-CM-06 | 25G00437-015 | 187 | 5,126,841 | 970,224 | 58 | 99.3 | 249,400 | 5 |
| WKUCMP2-E.coli-ESBL-D0-CM-01 | 25G00437-016 | 177 | 5,123,635 | 970,028 | 60 | 99.39 | 229,388 | 6 |
| WKUCMP2-E.coli-ESBL-D0-CM-02 | 25G00437-017 | 185 | 5,124,306 | 970,224 | 61 | 99.37 | 229,388 | 6 |
| WKUCMP2-E.coli-ESBL-D0-CM-03 | 25G00437-018 | 166 | 5,121,498 | 970,224 | 61 | 99.45 | 275,396 | 5 |
| WKUCMP2-E.coli-ESBL-D0-CM-05 | 25G00437-019 | 193 | 5,128,251 | 970,028 | 61 | 99.3 | 229,388 | 6 |
| WKUCMP2-E.coli-ESBL-D0-CM-06 | 25G00437-020 | 164 | 5,119,295 | 970,028 | 61 | 99.46 | 229,388 | 6 |

**Table S5: Antimicrobial resistance gene profiles detected by whole genome sequencing 20 isolates**

| Antimicrobial class | 515 (n=2) | 6416 (n=1) | 10 (n=3) | 10 (n=2) | 189 (n=2) | 2509 (n=10) |
|---|---|---|---|---|---|---|
| Aminoglycosides | *aph*(3")-Ib<br>*aph*(6)-Id | *aph*(3")-Ib<br>*aph*(6)-Id | *aph*(3')-Ia<br>*aac*(3)-IIe<br>*aad*A1 | *aph*(3")-Ib<br>*aph*(6)-Id | *aph*(3')-Ia<br>*aac*(3)-IId<br>*aad*A22 | *aph*(3")-Ib<br>*aph*(6)-Id<br>*aad*A5 |
| Beta-lactams | $bla_{CTX-M-1}$<br>$bla_{TEM-1}$ | $bla_{CTX-M-1}$<br>$bla_{TEM-1}$ | $bla_{CTX-M-55}$ | $bla_{CTX-M-32}$<br>$bla_{TEM-1}$ | $bla_{CTX-M-55}$<br>$bla_{TEM-1}$<br>$bla_{LAP-2}$ | $bla_{CTX-M-55}$ |
| Sulfonamides | *sul*2 | *sul*2 | *sul*3 | *sul*2 | *sul*3 | *sul*2 |
| Trimethoprim | | | | | *dfr*A14 | *dfr*A17 |
| Macrolides | *mph*(A) | *mph*(A) | | | *mph*(A) | |
| Lincosamide | | | | | *lnu*(F) | |
| Phenicol | *flo*R | *flo*R | *flo*R | *flo*R | *flo*R | *flo*R |
| Quinolones | *qnr*S1 | *qnr*B19 | *qnr*B19 | | *qnr*S1 | *gyrA*-D87N<br>*gyrA*-S83L<br>*par*C<br>*par*E |
| Tetracyclines | *tet*(A)<br>*tet*(M) | *tet*(A) | *tet*(A) | *tet*(A) | *tet*(A)<br>*tet*(B) | *tet*(A) |
| Fosfomycin | *glp*T<br>*uhp*T | *glp*T<br>*uhp*T | | | *glp*T | *glp*T |
| Rifamycin | | | | | *arr*-2 | |
| Nitrofurantion | | | | | | *nfs*A |
| Polymyxin | | | | | | *pmr*B |

**Table S6: Minimum inhibitory concentrations (MIC) by clinical interpretation and antimicrobial resistance gene profiles of 20 isolates analyzed by whole genome sequencing**

| | | Aminoglycosides | | | | | Quinolones | | | | |
|---|---|---|---|---|---|---|---|---|---|---|---|
| **Isolate_id** | **ST** | **Gen_mic** | **Gen_int** | **Str_mic** | **Str_int** | **Aminoglycoside resistance genes** | **Cip_mic** | **Cip_int** | **Nal_mic** | **Nal_int** | **Quinolone resistance genes** |
| 1 | 515 | 0.5 | S | =64 | R | *aph*(3")-Ib | 0.25 | S | 4 | S | *qnr*S1 |
| 2 | 6416 | 1 | S | =64 | R | *aph*(6)-Id | 0.5 | I | 16 | S | *qnr*B19 |
| 3 | 515 | 0.5 | S | 32 | R | | 0.12 | S | 4 | S | *qnr*S1 |
| 4 | 10 | >16 | R | 32 | R | *aph*(3')-Ia<br>*aac*(3)-IIe<br>*aad*A1 | 0.25 | S | 8 | S | *qnr*B19 |
| 5 | 189 | >16 | R | >64 | R | *aph*(3')-Ia<br>*aac*(3)-IId<br>*aad*A22 | 1 | R | 8 | S | *qnr*S1 |
| 6 | 10 | >16 | R | 16 | S | *aph*(3')-Ia<br>*aac*(3)-IIe<br>*aad*A1 | 0.5 | I | 16 | S | *qnr*B19 |
| 7 | 189 | >16 | R | >64 | R | *aph*(3')-Ia<br>*aac*(3)-IId<br>*aad*A22 | 0.5 | I | 8 | S | *qnr*S1 |
| 8 | 10 | 0.5 | S | =64 | R | *aph*(3")-Ib | ≤0.015 | S | 2 | S | None |
| 9 | | 1 | S | =64 | R | *aph*(6)-Id | ≤0.015 | S | 2 | S | detected |
| 10 | | >16 | R | 8 | S | *aph*(3')-Ia<br>*aac*(3)-IIe<br>*aad*A1 | 0.5 | I | 16 | S | *qnr*B19 |
| 11 | 2509 | 1 | S | >64 | R | *aph*(3")-Ib | >4 | R | >32 | R | *gyrA*-D87N |
| 12 | | 1 | S | >64 | R | *aph*(6)-Id | >4 | R | >32 | R | *gyrA*-S83L |
| 13 | | 1 | S | >64 | R | *aad*A5 | >4 | R | >32 | R | *par*C |
| 14 | | 0.5 | S | >64 | R | | >4 | R | >32 | R | *par*E |
| 15 | | 0.5 | S | >64 | R | | >4 | R | >32 | R | |
| 16 | | 0.5 | S | >64 | R | | >4 | R | >32 | R | |
| 17 | | 0.5 | S | >64 | R | | >4 | R | >32 | R | |
| 18 | | 1 | S | >64 | R | | >4 | R | >32 | R | |
| 19 | | 0.5 | S | >64 | R | | >4 | R | >32 | R | |
| 20 | | 0.5 | S | >64 | R | | >4 | R | >32 | R | |

| Isolate_id | ST | Amo_mic | Amo_int | Amp_mic | Amp_int | Fox_mic | Fox_int | Axo_mic | Axo_int | Mer_mic | Mer_int | Beta-lactamase genes detected |
|---|---|---|---|---|---|---|---|---|---|---|---|---|
| 1 | 515 | 8 | S | >32 | R | 4 | S | 32 | R | ≤0.06 | S | *bla*$_{CTX-M-1}$ |
| 2 | 6416 | 16 | I | >32 | R | 8 | S | 32 | R | ≤0.06 | S | *bla*$_{TEM-1}$ |
| 3 | 515 | 8 | S | >32 | R | 4 | S | 32 | R | ≤0.06 | S | |
| 4 | 10 | 4 | S | >32 | R | 4 | S | >64 | R | ≤0.06 | S | *bla*$_{CTX-M-55}$ |
| 5 | 189 | 8 | S | >32 | R | 16 | I | >64 | R | ≤0.06 | S | *bla*$_{CTX-M-55}$ *bla*$_{TEM-1}$ *bla*$_{LAP-2}$ |
| 6 | 10 | 16 | I | >32 | R | 4 | S | >64 | R | 0.12 | S | *bla*$_{CTX-M-55}$ |
| 7 | 189 | 8 | S | >32 | R | 16 | I | 64 | R | ≤0.06 | S | *bla*$_{CTX-M-55}$ *bla*$_{TEM-1}$ *bla*$_{LAP-2}$ |
| 8 | 10 | 8 | S | >32 | R | 4 | S | 64 | R | ≤0.06 | S | *bla*$_{CTX-M-32}$ |
| 9 | | 4 | S | >32 | R | 4 | S | 32 | R | ≤0.06 | S | *bla*$_{TEM-1}$ |
| 10 | | 4 | S | >32 | R | 4 | S | >64 | R | ≤0.06 | S | *bla*$_{CTX-M-55}$ |
| 11 | 2509 | 8 | S | >32 | R | 8 | S | >64 | R | ≤0.06 | S | *bla*$_{CTX-M-55}$ |
| 12 | | 8 | S | >32 | R | 8 | S | >64 | R | ≤0.06 | S | |
| 13 | | 8 | S | >32 | R | 8 | S | >64 | R | ≤0.06 | S | |
| 14 | | 8 | S | >32 | R | 8 | S | >64 | R | ≤0.06 | S | |
| 15 | | 8 | S | >32 | R | 8 | S | >64 | R | ≤0.06 | S | |
| 16 | | 8 | S | >32 | R | 8 | S | >64 | R | ≤0.06 | S | |
| 17 | | 8 | S | >32 | R | 8 | S | 64 | R | ≤0.06 | S | |
| 18 | | 8 | S | >32 | R | 8 | S | >64 | R | ≤0.06 | S | |
| 19 | | 8 | S | >32 | R | 8 | S | >64 | R | ≤0.06 | S | |
| 20 | | 8 | S | >32 | R | 8 | S | >64 | R | ≤0.06 | S | |

**Table S7: Functions associated with the virulence factor genes detected from the whole genome sequence of extended spectrum beta-lactamase producing *E. coli* isolated from dairy cattle manure.**

| Virulence factor | Protein function |
|---|---|
| AslA | arylsulfatase |
| F17A | Major fimbrial subunit of F17 |
| F17D | F17 Chaperone |
| F17G | Minor adhesive subunit of F17 fimbriae |
| anr | AraC negative regulator |
| capU | Hexosyltransferase homolog |
| cma | Colicin M activity |
| csgA | curlin major subunit CsgA |
| fdeC | intimin-like adhesin FdeC |
| fimH | Type 1 fimbriae |
| gad | Glutamate decarboxylase |
| hlyE | Avian E. coli haemolysin |
| hlyF | Hemolysin F |
| iss | Increased serum survival |
| iucC | Aerobactin synthetase |
| iutA | Ferric aerobactin receptor |
| lpfA | Long polar fimbriae |
| nlpI | lipoprotein NlpI precursor |
| ompT | Outer membrane protease (protein protease 7) |
| shiB | homologs of the Shigella flexneri SHI-2 pathogenicity island gene shiA |
| sitA | Iron transport protein |
| terC | Tellurium ion resistance protein |
| traJ | Protein TraJ (Positive regulator of conjugal transfer operon) |
| traT | Outer membrane protein complement resistance |

| | |
|---|---|
| yehA | Outer membrane lipoprotein, YHD fimbriael cluster |
| yehB | Usher, YHD fimbriael cluster |
| yehC | Chaperone, YHD fimbriael cluster |
| yehD | Major pilin subunit, YHD fimbriael cluster |
| yghJ | Metalloprotease |
